# Signatures of echolocation and dietary ecology in the adaptive evolution of skull shape in bats

**DOI:** 10.1038/s41467-019-09951-y

**Published:** 2019-05-02

**Authors:** Jessica H. Arbour, Abigail A. Curtis, Sharlene E. Santana

**Affiliations:** 1 Department of Biology, University of Washington, Seattle, WA 98195 USA; 2 Burke Museum of Natural History and Culture, University of Washington, Seattle, WA 98195 USA

**Keywords:** Evolutionary theory, Phylogenetics

## Abstract

Morphological diversity may arise rapidly as a result of adaptation to novel ecological opportunities, but early bursts of trait evolution are rarely observed. Rather, models of discrete shifts between adaptive zones may better explain macroevolutionary dynamics across radiations. To investigate which of these processes underlie exceptional levels of morphological diversity during ecological diversification, we use modern phylogenetic tools and 3D geometric morphometric datasets to examine adaptive zone shifts in bat skull shape. Here we report that, while disparity was established early, bat skull evolution is best described by multiple adaptive zone shifts. Shifts are partially decoupled between the cranium and mandible, with cranial evolution more strongly driven by echolocation than diet. Phyllostomidae, a trophic adaptive radiation, exhibits more adaptive zone shifts than all other families combined. This pattern was potentially driven by ecological opportunity and facilitated by a shift to intermediate cranial shapes compared to oral-emitters and other nasal emitters.

## Abstract (web summary)

What drives changes in morphological diversity? Here, Arbour et al. analyse skull 3D shape evolution across the bat radiation using µCT scan data, finding two phases of skull shape diversification, early adaptive shifts related to echolocation, and more recent shifts related to diet transitions.

## Introduction

Adaptive radiation, a process in which rapid species diversification occurs in tandem with morphological and ecological adaptation, is considered one of the major sources of phenotypic diversity across the Tree of Life^1–3^. Understanding the patterns and mechanisms leading to adaptive radiations, including how trait evolution influences biodiversity, is thus a major focus of evolutionary biology. Models predicting rates of speciation, morphological evolution, and ecological diversification^1,3,4^ suggest that most morphological disparity within adaptive radiations originates during an “early burst” of rapid species diversification related to niche filling. Empirical evidence further suggests that, indeed, maximum morphological disparity tends to be established early in the evolutionary history of many diverse clades (e.g., angiosperms, brachiopods, birds, cichlid fish, crinoids, among many others)^5–10^. However, systematic analyses of trait evolution rarely find significant evolutionary rate heterogeneity consistent with an adaptive radiation scenario (e.g., an early burst of trait evolution) at larger taxonomic scales (e.g., family or higher), with most major clades apparently evolving under strong selection^11^.

High early morphological diversity with a limited evolutionary rate heterogeneity across major radiations poses a paradox, because such patterns are unlikely to be generated under constant rate, random-walk evolutionary processes (e.g., Brownian motion, BM)^12–14^. Evolutionary biologists have tried to resolve this issue by using a framework of discrete adaptive events, including recently developed methods that allow testing for Simpsonian “adaptive zones” (i.e., shifts between discrete evolutionary transitions in morphology that, in turn, permit trait and species diversification within otherwise unexplored niche space)^9,15–17^. Testing the impact of these adaptive zone shifts on morphological and species diversity can be particularly informative if the evolution of ecologically-relevant morphological features is examined across large radiations. For example, in an extensive study of birds (>2000 species), Cooney et al.^9^ showed evolutionary rate heterogeneity in bill morphology that was predominantly restricted to branches with distinct shifts to novel, highly specialized bill shapes (e.g., pelicans and flamingos), whereas evolutionary rates in bill shape remained fairly consistent across other bird clades. Despite these advances, it remains unclear the extent to which such discrete macroevolutionary processes contributed to generating morphological diversity across most major vertebrate radiations.

Seemingly integrated anatomical structures may show varying levels of morphological diversity, and this may be driven, in part, by changes in either the rate or pattern of trait diversification^18,19^. For example, in an analysis across mammals, Linde-Medina et al.^18^ demonstrated variation in diversification potential both between the shape of the cranium and mandible, and between the shape of the rostrum and braincase. Similarly, an analysis of ground-squirrel skull characteristics found a “mosaic” of macroevolutionary processes impacting the evolution of different cranial traits^20^. The timing of trait diversification may also vary between different anatomical regions (e.g., head-first or tail-first models of fish body shape evolution^19^). To date, studies of adaptive zone shifts have primarily focused on a single trait or an overall shape metric^9,21^; however, adaptive shifts may vary across traits given differing constructional, ecological, or functional constraints.

Many important questions remain about the impact of adaptive zone shifts on diversification of anatomical structures, ecological guilds, and clades. For example, are Simpsonian adaptive zones a common theme in major vertebrate radiations? Do clades with exceptional ecological diversity exhibit a higher incidence of adaptive zone shifts^21,22^? And, do closely associated morphological structures show parallel shifts between adaptive zones, or are such macroevolutionary processes decoupled across traits^18,20^? We investigate these questions while focusing on one of the most morphologically diverse and functionally important structures in the vertebrate body, the skull, and the most ecologically diverse mammalian radiation, bats (Order Chiroptera)^23–25^. Although large-scale phylogenetic comparative analyses are lacking, skull morphological diversity in bats has been linked to variation in diet^24–26^, feeding performance^27^, and primary sensory mode^28–30^, which is representative of trends in other mammals. Furthermore, the evolution of bat skull traits has been linked to an expansion into a multitude of dietary niches and subsequent species diversification within New World leaf-nosed bats (Phyllostomidae)^23,25,31^, whereas other species-rich clades, such as horseshoe bats (Rhinolophidae) and evening bats (Vespertilionidae) appear to be morphologically uniform. Therefore, phylogenetic explorations of this system have exceptional potential to illuminate whether and how evolutionary rate shifts are integrated between morphology and ecology during clade diversification.

Major and distinct ecological transitions occurred during the early evolution of bats (e.g., evolution of laryngeal echolocation and subsequent loss in Pteropodidae, the second largest bat family), as well as during the radiation of some New World clades (e.g., numerous dietary shifts among Phyllostomidae). Consequently, we hypothesize that the dynamics of skull shape macroevolution in bats are better described by a series of discontinuous shifts that correspond to innovations in dietary ecology and sensory adaptations, rather than a model of evolutionary rate heterogeneity. Due to developmental, functional, and ecological constraints on skull shape in bats^25,27,29,32–36^, we predict that adaptive shifts will be shared across the cranium and mandible. We also predict that (1) adaptive zones corresponding to major sensory modes will differ in cranial morphology (e.g., the orientation of the rostrum between oral and nasal echolocators^30^), and (2) increased ecological diversification in Phyllostomidae is tied to increased adaptive shifts in skull shape evolution, particularly with respect to shape changes that are linked to bite performance and the mechanical demands of different diets (e.g., rostral length and cranium height)^36^^,^^37^.

To test these predictions, we assembled a comprehensive dataset describing the 3D shape of the cranium and mandible across 203 species of bats. We employed multivariate comparative phylogenetic approaches to address adaptive landscape dynamics across the primary axes of skull shape disparity. Morphological diversity of the cranium and mandible was established early, but skull shape evolution is better described by a series of adaptive shifts rather than an early burst of evolution. Early adaptive shifts are associated with adaptations for echolocation across bats, while later and more numerous shifts are associated with dietary niche transitions in Phyllostomidae. The evolution of skull shape disparity in bats has potentially been driven by adaptive zone shifts modulated by ecological opportunity and prior adaptations.

## Results and Discussion

### Chiropteran skull shape disparity

**Fig. 1:**
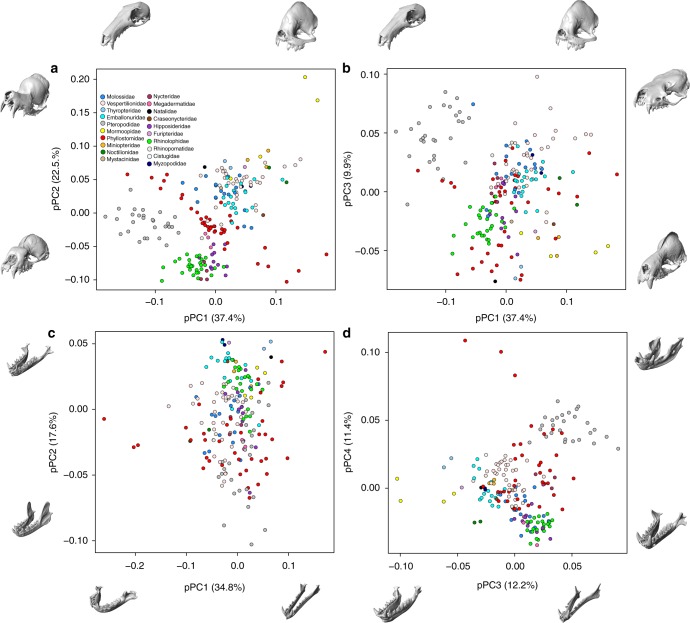
Cranial and mandibular morphospaces of bats based on phylogenetic PCA. Digital models of crania and mandibles from μCT data illustrate the trends across each axis, 3D models visualized using Checkpoint. Taxa illustrated: Cranium (**a**, **b**) —pPC1 (+*Centurio senex*, −*Macroglossus sobrinus*), pPC2 (+*Mormoops blainvillei*, −*Hipposideros caffer*), pPC3 (+*Tylonycteris robustula*, − *Lophostoma silvicolum*); Mandible (**c**, **d**) —pPC1 (+*Choeronycteris mexicanus*, −*Centurio senex*), pPC2 (+*Myzopoda aurita*, −*Dobsonia praedatrix*), pPC3 (+*Macroglossus sobrinus*, −*Mormoops megalophylla*), pPC4 (+*Desmodus rotundus*, −*Megaderma spasma*). Percent values give the *R*^2^ from a Procrustes multiple regression of landmark coordinates on pPC scores, see Methods and source data file

**Fig. 2:**
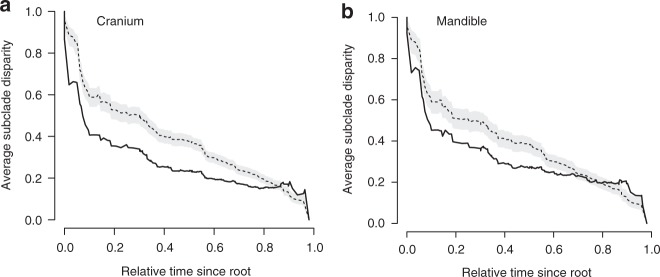
Disparity-through-time plots for the shape of the cranium and mandible across bats. Thick black line = observed subclade disparity. Gray polygon = area of 95% confidence interval of BM simulated character histories. Dashed line = medium DTT curve of BM simulated character histories. See source data file

To investigate skull shape disparity and macroevolutionary patterns across bats, we first summarized variation in cranial and mandibular shape using multivariate, 3D geometric morphometric methods that incorporated evolutionary relationships among species. After accounting for the impact of phylogenetic relationships, the cranial shape of 202 bat species varied primarily along three phylogenetic Principal Components (pPC), explaining a total of 69.8% of absolute shape variation: pPC1–anteroposterior cranial elongation, pPC2–dorso-ventral rostral flexure, and pPC3–cranial height (Fig. 1). Even with phylogenetic correction, morphological disparity in cranial shape was still strongly structured by early divergence events, especially along pPC1 and pPC2, with the suborder Yinpterochiroptera (non-echolocators and primarily nasal echolocators) showing crania with relatively long, ventrally flexed rostra. Within the Yinpterochiroptera, this trend was especially marked among Pteropodidae, but with the exception of *Craseonycteris* (Figs. 1 and 2). The suborder Yangochiroptera (ancestrally oral-emitters, with nasal-emitters within Nycteridae and Phyllostomidae) showed comparatively shorter, more dorsally flexed rostra, with the exception of Nycteridae, which overlapped in shape space with Rhinolophidae and Hipposideridae across all three axes (Fig. 1). Phyllostomidae exhibited remarkable cranial diversity and overlapped with the shape space of nearly all other lineages across pPC axes, except with the strongly dorsiflexed rostrum in *Mormoops* and extremely dorsiventrally-flattened crania of some Vespertilionidae, Molossidae, and Pteropodidae (Fig. 1). Interestingly, while there was substantial overlap in cranial shape among Yangochiropteran families (especially across primarily oral-emitting clades like Vespertilionidae, Emballonuridae, and Molossidae), phyllostomids occupied an intermediate morphospace across pPC1 and pPC2 between other Yangochiropterans and the Yinpterochiroptera (Fig. 1).

Mandibular shape of 191 bat species varied primarily along four major pPCs after phylogenetic correction, explaining 76.0% of absolute shape variation: pPC1—anteroposterior mandible elongation, pPC2—mandible body and coronoid height, pPC3—dorsiventral mandible flexure, and pPC4—tooth row length relative to the length of the mandible body (Fig. 1). Bat families showed considerably more overlap in mandibular shape space compared to the cranium. Again, phyllostomids showed considerable disparity, with extreme morphologies (high or low pPC scores) in nectar feeders (e.g., *Choeronycteris*: narrow/elongate mandible), durophagous frugivores (e.g., *Centurio*: u-shaped mandible) and sanguinivorous bats (*Desmodus*, *Diphylla*, *Diaemus*: short, anteriorly positioned tooth row; Fig. 1). Families dominated by insectivorous lineages from both Yinpterochiroptera and Yangochiroptera (Supplementary Fig. 8) overlapped in mandible shape space, sharing moderately narrow, dorsally flexed mandibles with low coronoid processes and a long tooth row, especially the post-canine dentition (Fig. 1). The pteropodids primarily exhibited ventrally flexed mandibular bodies relative to the ramus, a short tooth rows and tall coronoids.

**Fig. 3:**
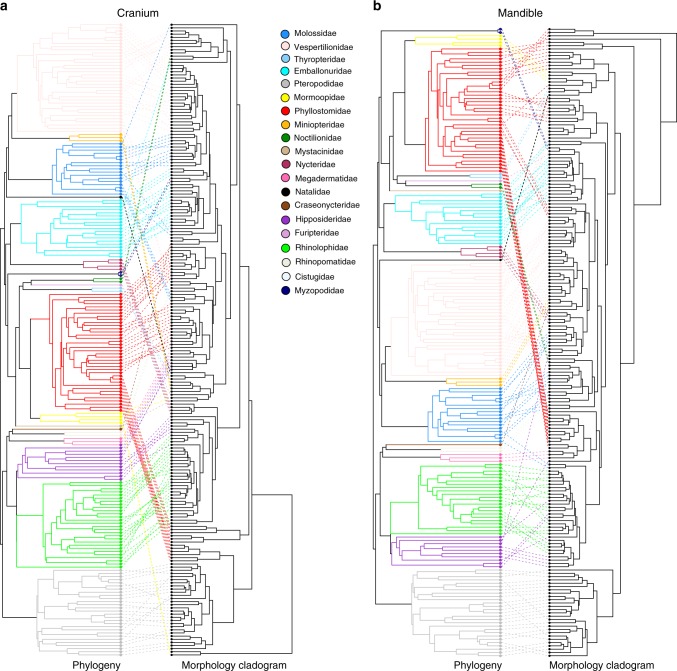
Tanglegrams of phylogenetic relationships of Chiroptera, and morphology dendrograms of bat skull shape. **a** cranium, **b** mandible. Dotted lines link the same species in both trees

We tested whether cranial and mandibular disparity was established early in the evolution of bats, a pattern often attributed to declining evolutionary rates^3,8,13,14^. Analyses of 3D cranial and mandibular morphological variation revealed a pattern of early shape divergence, with disparity focused between rather than within early lineages (low average subclade disparity). Average subclade shape disparity dropped early during the radiation of bats for both the cranium and mandible (Fig. 2), with MDI values differing significantly from expectations under BM evolution (cranium—MDI = −0.108, *p* < 0.001; mandible—MDI = −0.0647, *p* < 0.001). Morphology–phylogeny tanglegrams of the bat cranium and mandible showed strong correspondence between shape disparity and evolutionary relationships (Fig. 3), compared to the random walk evolutionary expectation (Supplementary Fig. 3). The observed taxon displacement between the phylogeny and morphology-based dendrograms was significantly less (cranium tip displacement value = 17.9, *p* = 0.015; mandible tip displacement value = 17.1, *p* = 0.002) than that expected under a multivariate BM process (cranium simulated values = 14.8–52.9; mandible simulated values = 15.0–53.4). Simulated (under BM) tanglegrams showed a higher incidence of mismatches between the phylogeny and morphological relationships (e.g., more steep lines; Supplementary Fig. 4) than our observed datasets (Fig. 3). Within most families, skull morphology was more strongly conserved than expected under purely random-walk evolution, especially within Pteropodidae (Fig. 3). Taxa showing relatively larger morpho-phylogenetic mismatches included phyllostomid nectar feeders, sanguinivorous bats, and durophagous frugivores, with an additional division in mandible shape between predominantly animalivorous and predominantly frugivorous clades. Other clades showing high morphology–phylogeny discordance included species from Rhinolophidae, Hipposideridae, flat-headed vespertilionids, and small or poorly sampled families (*n* < 4 species; Fig. 3).

Overall, bat skull disparity was strongly impacted by early divergence events and predominantly preceded the establishment of modern chiropteran families, especially those with high diversity. Phyllostomidae showed the greatest departure from these early trends in morphological diversity.

### Bat skull evolution has undergone adaptive zone shifts

**Fig. 4:**
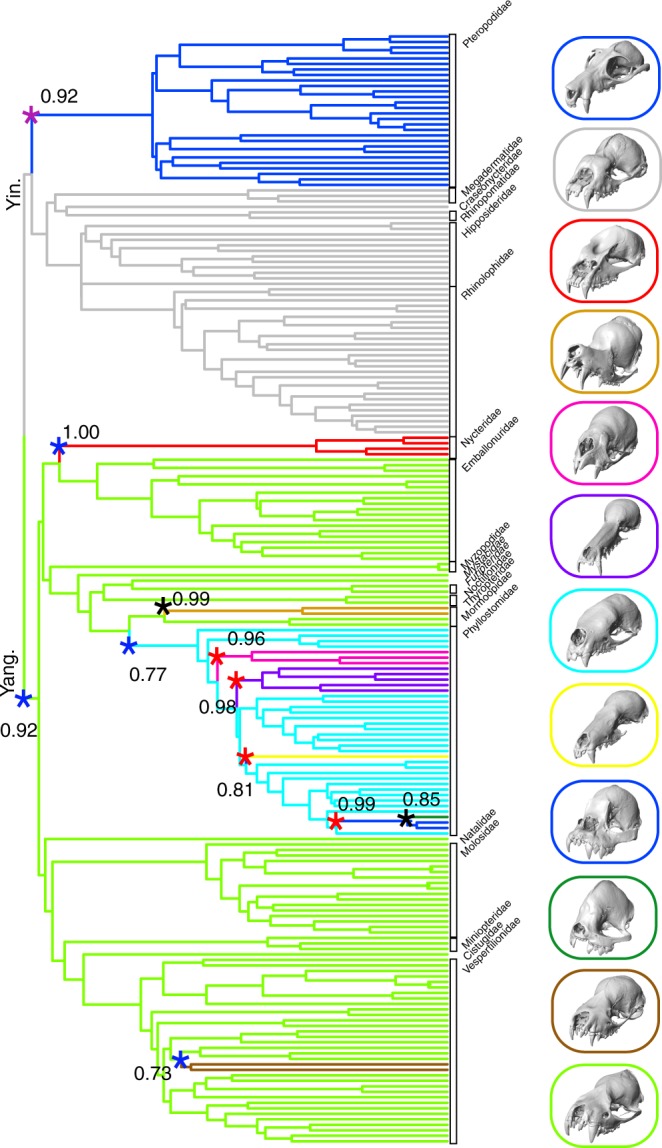
Evolutionary shifts (asterisks) in cranium shape across bats. Shifts were determined by l1ou adaptive landscape model fitting on pPCA scores using pBIC (pPC 1–3; Fig. 1). Bootstrap support is given for shift locations. Blue shifts = transition in echolocation type, Red shifts = transitions in diet, Purple shifts = transition in echolocation type and diet, Black shifts = no transition. Digital models of crania from μCT data illustrate the sample taxa from each adaptive regime, 3D models were visualized using Checkpoint. Representative taxa, from top to bottom: *Pteropus poliocephalus, Hipposideros caffer, Nycteris hispida, Mormoops blainvillei, Desmodus rotundus, Choeronycteris mexicana, Phylloderma stenops, Ametrida centurio, Centurio senex, Corynorhinus townsendii, Murina leucogaster*. Yin. = Yinpterochiroptera. Yang. = Yangochiroptera

**Fig. 5:**
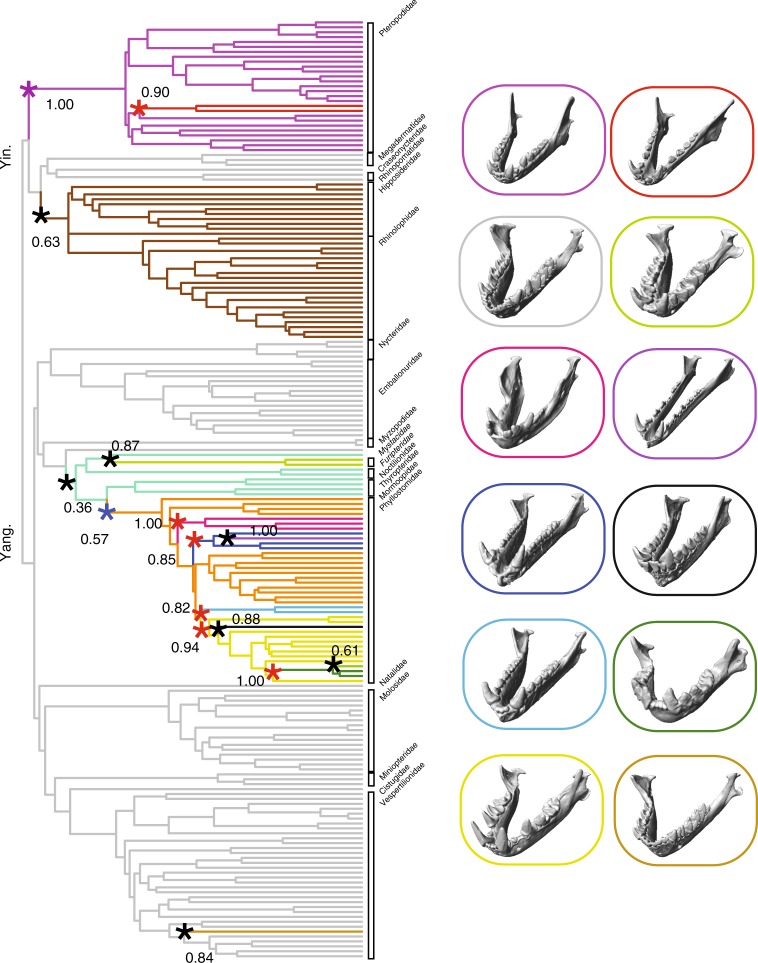
Evolutionary shifts (asterisks) in mandible shape across bats. Shifts were determined by l1ou adaptive landscape model fitting on pPCA scores using pBIC (pPC 1–4; Fig. 1). Bootstrap support is given for shift locations. Blue shifts = transition in echolocation type, Red shifts = transitions in diet, Purple shifts = transition in echolocation type and diet, Black shifts = no transition. Representative taxa from well-supported shifts, from top to bottom; Left: *Rousettus aegyptiacus, Myotis lucifugus, Desmodus rotundus, Anoura geoffroyi, Lonchophylla robusta, Chiroderma villosum*; Right: *Syconycteris australis, Noctilio leporinus, Choeronycteris mexicana, Rhinophylla pumilio, Sphaeronycteris toxophyllum, Tylonycteris robustula*. Yin. = Yinpterochiroptera. Yang. = Yangochiroptera

To determine if skull shape has undergone Simpsonian adaptive shifts in bats, we used a model fitting algorithm implemented in the R package l1ou^38^, which estimates shifts between selective regimes and requires no prior hypotheses regarding locations of shifts. We selected the best-fit shift configurations using phylogenetic Bayesian Information Criterion (pBIC, see Methods). Across all bats, we found extensive adaptive evolutionary shifts in the morphological evolution of both the cranium (11 shifts) and mandible (15 shifts) (Figs. 4 and 5; Supplementary Table 2). All cranial shifts showed moderate to strong bootstrap support (>70%), while several mandibular shifts were poorly supported (<70%), including those at the bases of Rhinolophidae + Hipposideridae, Phyllostomidae, Noctilionidae, and *Centurio*, respectively (Figs. 4 and 5).

The adaptive evolution of cranial shape across bats can be chiefly described by two discrete patterns; first, early (~58–34 Ma) adaptive evolutionary shifts across bats, and more recent (~26 Ma to present) shifts primarily within the Phyllostomidae (see discussion below). Early adaptive shifts in cranial shape (as represented by pPC scores) were found at the base of Yangochiroptera, the base of Pteropodidae, Mormoopidae, Nycteridae, and two sister Vespertilionidae genera, *Corynorhinus* and *Plecotus* (Fig. 5). Evolutionary shifts in mandible shape were identified at the base of and within Pteropodidae (*Syconycteris* + *Macroglossus* clade, nectarivores), at the base of Noctilionidae, leading to the vespertilionid genus *Tylonycteris*, as well as within phyllostomids (Fig. 5, and see discussion below). To determine if adaptive zone shifts better describe skull evolution in bats than simpler evolutionary processes (e.g., a constant rate, random walk, BM; an early burst of evolution, EB; constant rate under selective constraint, OU), we fit fully multivariate evolutionary models that contrasted adaptive landscapes estimated by l1ou with other models (BM, OU1, EB). We found that a model incorporating adaptive zone shifts had stronger support than simple evolutionary models (BM, OU1) as well as the EB model, which is used to describe a decrease in evolutionary rates through time and is frequently attributed to clades with early morphological diversity^8,11,13^. Model fitting within Phyllostomidae paralleled the results for Chiroptera (support for l1ou model over the dietary model and BM/OU1/EB models; Supplementary Table 3).

The majority of cranial adaptive shifts across bats were supported regardless of whether or not phylogenetic correction was incorporated into the PCA (Supplementary Fig. 13). The largest difference in the results for the cranium was the position of a shift corresponding to most nasal echolocators in Yinpterochiroptera, which excluded Megadermatidae and Rhinopomatidae in the PCA results. The impact of PC score phylogenetic correction on adaptive shift estimation and model fitting was greatest for the mandible dataset. Mandible morphology across bats varied much more strongly with phylogenetic relationships when standard PCA was implemented (Fig. 1 vs. Supplementary Fig. 12). While the majority of shifts from the PCA analyses were consistent with the pPCA results, including shifts within sanguinivores, durophagous frugivores, some nectarivores and generalist frugivores, PCA resulted in substantially fewer reconstructed adaptive shifts overall (Supplementary Fig. 14). However, the patterns from macroevolutionary model fitting remained the same in both analyses (i.e., high model support for adaptive shifts than simple evolutionary models; Supplementary Table 4). Results across pPCA and PCA for phyllostomids-specific analyses were also largely equivalent (Supplementary Figs. 15–16, Supplementary Table 5).

### Adaptive shifts are linked to ecological diversity in bats

For both the cranium and mandible, numerous evolutionary shifts were concentrated within the most trophically-diverse bat family, Phyllostomidae (6 of 11 cranial shifts and 9 of 15 mandibular shifts in morphospaces based on pPCA scores; Figs. 4 and 5). Well-supported shifts in cranial shape evolution were found at the base of Desmodontinae (sanguinivores), Glossophaginae and Lonchophyllinae (nectarivores), and within the Stenodermatinae clade of *Centurio* + *Ametrida* + *Sphaeronycteris* (durophagous frugivores; and see Supplementary Fig. 6 for subfamilies).

**Fig. 6:**
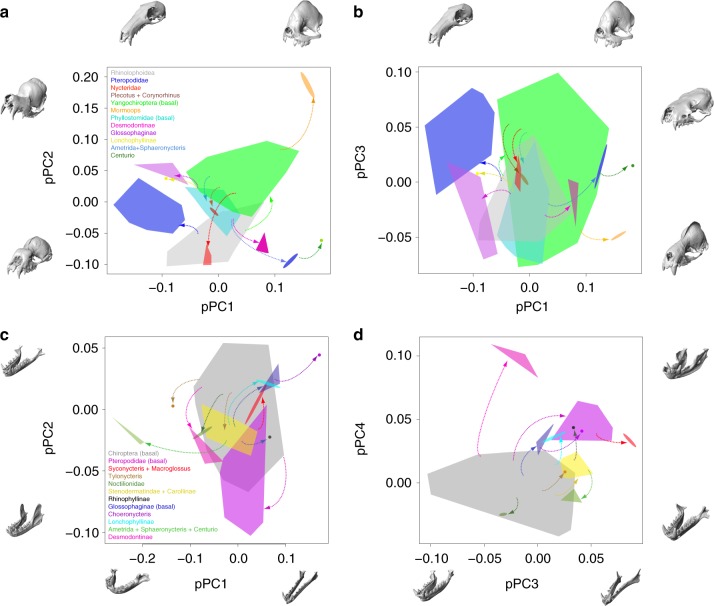
Adaptive shifts in cranial and mandibular morphospaces (pPC scores). Digital models of crania and mandibles from μCT data illustrate the trends across each axis, 3D models were visualized using Checkpoint. Illustrated taxa as in Fig. 1. Polygons indicate pPC score distributions of all species representing an adaptive evolutionary regime shift for the cranium (**a**, **b**) or mandible (**c**, **d**) with at least 0.7 bootstrap support. Arrows indicate the transition between ancestral adaptive regime (gray polygon) and the new adaptive regime for each evolutionary shift. Colors of adaptive regimes match those illustrated in Figs. 4 and 5. See source data file

Glossophagines and lonchophyllines were both characterized by shifts that enabled movement into morphospace areas representing long and narrow skulls (particularly *Choreonycteris*; Figs. 4–6), which accommodate the long tongues necessary for nectar-feeding. The morphologies of these skulls—a long jaw outlever and relatively smaller regions for jaw adductor muscle attachments—would also result in relatively low mechanical advantage and bite forces^37^, which are consistent with the liquid diet of these species. In sharp contrast, durophagous frugivores such as *Ametrida*, *Centurio*, and *Sphaeronycteris*, radiated into morphospaces characterized by short rostra, wide u-shaped mandibles and robust crania (Figs. 4–6). These traits have been previously associated with high mechanical advantage and high bite forces^24,27,37,39^, which allow for the consumption of hard fruit and seeds^40^. Shifts associated with sanguinivory resulted in a moderately wider skull (both cranium and mandible), a shorter rostrum, and a mandible with a short tooth row (Fig. 6).

When l1ou was carried out on a phyllostomid-focused phylogeny and morphospace, we found these same shifts (except within Glossophaginae), plus a shift separating the frugivorous clade (Stenodermatinae + Rhinophyllinae + Carolliinae) from animalivorous/omnivorous taxa (Supplementary Figs. 10–11). Well-supported shifts in mandible shape evolution were also found within Phyllostomidae. These coincided with several cranial shifts, including those detected in nectarivores (basal within Glossophaginae as well as *Choeronycteris* and Lonchophyllinae), durophagous frugivores, and sanguinivores. Additional shifts in mandible shape evolution separated frugivorous and animalivorous/omnivorous species, and a shift leading to *Rhinophylla* (Fig. 5). By comparison, analyses using a phyllostomid-focused phylogeny did not find separate shifts for nectarivores but did find a shift separating plantivorous and animalivorous taxa (Supplementary Figs. 10–11). Where shifts in skull shape separated these dietary groups, animalivorous taxa possessed comparatively taller braincases (often high sagittal crests), a longer tooth row, and more robust mandibles (taller mandible body, Supplementary Figs. 10–11).

Overall, the recovered adaptive zone shifts within Phyllostomidae were strongly associated with shifts in dietary niches, especially to novel food resources such as nectar, blood, and hard fruits (Figs. 4 and 5; Supplementary Figs. 10–11). Previous studies have demonstrated that dietary hardness had a significant impact on the diversification of feeding biomechanics in phyllostomids^24,25^. Rossoni et al.^33^ similarly observed strong natural selection on skull shape associated with adaptation to and specialization for novel dietary resources within phyllostomids. While adaptive landscapes generated through l1ou were largely concordant with these dietary categories, they did not differentiate among animalivorous diets (insectivory, carnivory, and omnivory; Figs. 4 and 5, Supplementary Figs. 7–8). Consistent with this finding, previous morphometric comparisons among animalivorous bats found substantial overlap in skull shape between vertebrate-eaters and other animalivorous taxa (except the limited number of piscivorous species)^26^. Rather, some animalivorous diets were associated with the evolution of larger body size and allometry-driven changes in skull shape^26^. Adaptations to carnivory may also involve dental or myological characters^28^.

### Shifts do not occur in parallel across skull parts

The observed adaptive shifts patterns (Figs. 4 and 5) suggest a decoupling of primary ecological constraints on shape evolution between the cranium and mandible in bats. This result was further supported by tests of multivariate, evolutionary models contrasting dietary and echolocation transitions; across both structures, 1lou adaptive landscape models were generally better supported than models representing dietary transitions or echolocation type alone (Table 1). However, while neither the diet model nor the echolocation type model were the best fit to our data, we did find greater support for the echolocation type model than the dietary model for cranial shape, but the reverse for mandibular shape (Table 1). Interestingly, l1ou results based on standard PCAs of mandible shape (which showed fewer shifts than the pPCA result) showed much greater similarity in fit to the dietary model than pPCA (minimum ΔAICc 14.72 vs. 108.78 respectively; Tables 1 and S4).

**Table 1:** Results of mvMorph multivariate model fitting of cranium and mandible shape evolution across bats

Structure	Model	Regimes	Number of parameters	Log likelihood^a^	AICc^a^	ΔAICc^a^
Cranium	l1ou	12	45	−1333.007	2763.41	0
	OU-EM	3	21	−1423.71 (−1429.64, −1420.37)	2891.00 (2884.32, 2902.86)	127.59 (120.91, 139.45)
	OU-DIET	6	30	−1424.98 (−1447.40, −1418.96)	2915.19 (2901.59, 2958.03)	151.78 (138.18, 194.62)
	EB	1	10	−1456.83	2934.03	170.62
	BM	1	9	−1476.06	2970.42	207.01
	OU1	1	15	−1476.06	2982.93	219.52
Mandible	l1ou	16	80	−1386.46	2951.89	0
	OU-DIET	6	44	−1490.70 (−1581.76, 1483.69)	3074.91 (3060.67, 3257.02)	123.02 (108.78, 305.13)
	OU-EM	3	32	−1576.18 (−1580.70, −1574.66)	3219.26 (3216.2, 3228.29)	267.37 (264.31, 276.4)
	EB	1	15	−1617.4	3264.88	312.99
	BM	1	14	−1631.4	3291.4	339.51
	OU1	1	24	−1626.32	3302.26	350.37

*BM* Brownian motion, *OU* Ornstein–Uhlenbeck, *EB* early burst, *l1ou* adaptive landscape model from l1ou

^a^Multipeak OU models for echolocation emission type (EM) and dietary group (diet) were summarized over a distribution of 100 stochastic character mapping reconstructions, results are given as median (min, max)

Early adaptive shifts in cranial shape evolution (based on pPCA scores, Fig. 4 and Supplementary Figs. 6–7) match (1) a division between the laryngeal-echolocators, representing most bat families, and non-laryngeal echolocators in Pteropodidae, and (2) subsequent shifts within laryngeal echolocators (oral and nasal emitters). These macroevolutionary trends in cranial shape are concordant with the hypothesized single origin of laryngeal echolocation in bats and subsequent loss in Pteropodidae, the multiple origins of nasal-emission in Rhinolophoidea, Nycteridae, and Phyllostomidae, as well as the facultative nasal emission in some vespertilionid bat genera^29,41^. Early shifts in cranial shape were associated with nearly complete divergence in morphology along two major axes variation (elongation and flexure; Fig. 1 and Supplementary Fig. 7). Non-echolocators tended to exhibit a combination of more elongate and dorso-ventrally flat crania than echolocating bats (Supplementary Fig. 7). Comparatively, cranial shape differentiation within laryngeal echolocators was chiefly driven by rotation of the rostrum relative to the basicranium, with nasal emitters showing ventral flexion of the rostrum compared to oral emitters of similar cranial elongation (Fig. 4 and Supplementary Fig. 7). Differences in rostral flexion relative to the basicranial axis may allow echolocating bats to direct call beams in the line of flight without adjusting head position^28,30,32^. Dorsiflexion of the rostrum in oral echolocators aligns the gape more anteriorly, allowing calls to be transmitted directly ahead of the bat in flight^30^. Conversely, ventral rotation of the rostrum compared to the basicranium aligns the nostrils directly forward. Ventral flexion of the rostrum (pPC2) was greatest in the Rhinolophidae, Hipposideridae, Megadermatidae, and Nycteridae (on both pPC and PC scores; Fig. 1 and Supplementary Fig. 12), all of which possess considerable cranial modifications associated with nasal-emission. For example, both Rhinolophidae and Hipposideridae have enlarged, dome-shaped nasal cavities with a comparably large nasal apertures^28,30,42^, and show the most extreme rotation of the rostrum compared to the basicranium^28^ (Fig. 1 and Supplementary Fig. 12).

Constructional constraints stemming from rostral dorsiflexion likely impact other axes of cranial shape variation, and may result in considerable functional tradeoffs on other aspects of ecological adaptation. For example, it is likely that very elongate rostra can only be upturned so much without compromising feeding performance, especially for gleaning species. The region of morphospace where the rostrum would be most dorsiflexed showed few elongate skulls (under both PCA and pPCA, Fig. 1 and Supplementary Fig. 12), whereas crania with the most dorsiflexed rostra belong to species with shortened skulls (*Mormoops*). Additionally, certain dietary niches are largely or completely restricted to nasal echolocators or non-echolocators, such as carnivory and nectarivory, and these habits are generally associated with long, narrow skulls (Supplementary Fig. 9, with the exception of piscivores like *Noctilio*).

In contrast with patterns of cranial evolution, most well-supported adaptive shifts in mandible shape evolution were associated with dietary transitions, especially within Phyllostomidae; all three echolocation types show substantially greater overlap in mandible than in cranial shape (Supplementary Fig. 7). Only a single evolutionary shift in mandibular shape evolution was potentially associated with a transition between echolocation types, separating laryngeal echolocators from pteropodids (Fig. 5). However, this shift was also potentially tied to a transition in diet among the plantivorous Pteropodidae (Supplementary Figs. 6–9). Across nearly all bat families, adaptive zone shifts were largely decoupled across the cranium and mandible, however these structures underwent several parallel shifts related to feeding ecology in Phyllostomidae. Adaptive zone shifts co-occur across both structures within at least sanguinivores (Desmodontinae), nectarivores (Glossophaginae and Lonchophyllinae), durophagous frugivores (*Centurio, Ametrida*, and *Sphaeronycteris*), and potentially within all phyllostomid frugivores (Figs. 4 and 5; Supplementary Figs. 10–11).

Previous studies have suggested that echolocation may experience trade-offs with other sensory modalities such as olfaction and vision among nasal-emitting taxa, with families like Rhinolophidae evolving skulls more efficient at sound transmission and Phyllostomidae maintaining a larger olfactory skeleton within the rostrum, and larger eyes^29,30^. Morphologically, Phyllostomidae cranial shape spanned the entire range of skull elongation (pPC1 and PC1/PC2), but exhibited rostral flexion intermediate between other nasal echolocators and oral echolocators (when comparing similarly elongate skulls; Fig. 1 and Supplementary Fig. 12). Another adaptive shift in cranial shape (albeit recovered only from pPCA scores and not PCA scores; Figs. 4 and S13) towards this intermediate cranial morphospace also included species in *Corynorhinus* + *Plecotus* (Fig. 6) genera, which are known to vary between oral and nasal echolocation to some degree. The preceding shifts to a moderate laryngeal echolocator morphospace may have relaxed constraints on cranial shape evolution associated with echolocation, and facilitated a greater integration in cranium and mandible shape evolution in Phyllostomidae. Adaptive zone shift dynamics may therefore vary not only across anatomical structures, but may also be dependent upon prior morphological or ecological adaptations^43^.

### Detecting adaptive shifts in multidimensional shape data

While macroevolutionary analyses of adaptive evolution in morphology and function have undergone significant advances in recent years^21,22,44,45^, numerous methodological issues and limitations remain in the use of highly multidimensional shape data such as geometric morphometric landmark configurations (where each landmark is itself a multidimensional trait)^46^. While geometric morphometric approaches may be better equipped to describe complex shape variation by preserving the position of structures in their original shape space—as opposed to linear morphometrics or similar approaches—the size of these datasets presents unique concerns. First, the number of free parameters in multivariate macroevolutionary models (BM, OU, EB, etc.) increases exponentially with the number of traits, which is particularly egregious for large and complex shape datasets. Varied computational trade-offs have been made to apply such adaptive shift estimation approaches to large shape datasets. For example, compared to l1ou used here^38^, the recently released PhylogeneticEM package in R takes the alternate approach of ignoring differences in selective constraint across traits in favor of retaining trait correlations in model fitting. Other search algorithms, such as the Bayesian estimation approach of bayou, are (as of yet) only for univariate trait data, but may allow for more direct comparisons with alternate evolutionary models and better address issues of identifiability of shift positions^44^.

Second, whether trait reduction for such analyses is appropriate, and how model assumptions under phylogenetic corrections may impact macroevolutionary model fitting remain hotly debated issues^47,48^. While some approaches have been developed to calculate evolutionary rates across all traits in a multivariate dataset simultaneously, none of these can so far accommodate non-BM evolution or shifts across a phylogeny^46,48^. Lastly, the ability to incorporate phylogenetic uncertainty (in topology or branch lengths) by iterating such analyses over some distribution of trees (e.g., the posterior distribution of trees from a Bayesian phylogenetic analysis) or alternate phylogenetic analyses (focusing on different taxonomic levels), is computationally intensive and not frequently addressed.

Our selection of methods for the reduction of trait dimensions (pPCA and PCA), computational trade-offs (e.g., the use of l1ou), and phylogenetic uncertainty (phylogenies from all bats or family specific trees), are all likely to bias our results. Analyses with other methods could find some differences in the position of adaptive shifts in cranium and mandible shape in bats. However, we believe the major patterns highlighted in our results will be robust to other methodologies. Firstly, the relative impacts of selection on skull shape diversification for echolocation and diet vary between the cranium and mandible in bats. Secondly, phyllostomids appear to experience a higher frequency of adaptive shifts in skull shape evolution than any other bat family examined. Lastly, the most consistently recovered shifts in phyllostomids and some other groups like Pteropodidae were in clades exploiting functionally novel resources (specifically, blood feeding in Desmodontinae, nectar feeding in Glossophaginae and Lochophyllinae, and hard fruit in *Ametrida* + *Centrurio* + *Sphaeronycteris*). These patterns were recovered across multiple phylogenies, with both standard and phylogenetically-corrected PCA, and with both adaptive shift estimation and trait mapping approaches.

### Patterns in the evolution of bat skull shape

Previous comparative analysis of lineage diversification across the global radiation of bats revealed that speciation rates were high during the early evolution of this group, and then slowed through time (excepting Stenodermatinae within Phyllostomidae^23,25^). We found that morphological disparity was partitioned early in chiropteran evolution (Fig. 2), with strong morphological conservatism within modern families and limited convergence across lineages (Fig. 3). Concomitantly, early transitions in skull shape evolution were critical to modern morphological disparity (Figs. 4–6 and Supplementary Figs. 6–9). While the early establishment of morphological diversity is often attributed to an early burst of evolution^8,11,13,14^, multivariate model fitting found poor support for evolutionary rate heterogeneity over both an adaptive landscape generated by l1ou and for adaptive peaks representing dietary or sensory ecology. Rather, our results point to transitions between “Simpsonian” adaptive zones as a more appropriate theoretical framework for understanding evolution of morphological diversity in bats^15,17^. Comparative analyses in other vertebrate radiations (e.g., birds, canids, cichlid fish, etc.) have also stressed the importance of such discrete adaptive transitions on trait evolution^9,17,49^ and highlight the potential sparsity of “early burst” processes in morphological diversification^11^.

The common ancestor of modern bats is hypothesized to have been a small-bodied, laryngeal echolocating insectivore^29^. Size-driven constructional constraints combined with the pressure for specific degrees of rostral flexure among early laryngeal echolocators may have limited the potential for adaptive shifts in skull shape relating to dietary adaptations. Later evolution of larger body size among echolocators, or reduced selective pressure on echolocation performance^29,30^, may have provided an opportunity for new adaptive shifts in skull shape evolution in some clades. In particular, the heightened diversification of New World phyllostomid skulls and adaptation to a wide variety of novel dietary niches may have been facilitated by such prior adaptive shifts related to sensory ecology. Novel ecological opportunities in the Neotropics may have permitted this increase in adaptive zone shifts and facilitated extensive ecological and morphological radiation.

## Methods

### Phylogenies

For comparative analyses and phylogenetic corrections, we used a pruned version of a recent molecular phylogenetic analysis of Chiroptera^23^, which represents one of the most comprehensively sampled multi-locus phylogenies available for this order. However, while this phylogeny sampled widely across bats and is congruent with major relationships across bat families from previous phylogenetic analyses, some sub-family relationships (e.g., within Phyllostomidae) were at times weakly supported or inconsistent with some previous assessments^25,50,51^. This is likely a result of a high degree of missing data. As Phyllostomidae is of particular interest for adaptive evolutionary shifts in skull morphology^24,25^ (and see Results and discussion), we used a subtree of Noctilionoidea that densely sampled taxa from Phyllostomidae^51^ for additional analyses on this group. This phylogeny represented a fully Bayesian phylogenetic estimation and divergence time analysis, and also included additional fossil calibrations within Phyllostomidae.

### CT scanning and geometric morphometrics

Our comparative sample spanned 203 species of bats (202 species with cranial data, 191 species with mandibular data; *n* = 1–8 skulls per species) representing all currently established families and >50% generic coverage^23,52,53^. We scanned skulls using a Skyscan 1172 uCT scanner (Skyscan, Belgium) at the University of Washington, Seattle, WA, and a GE Phoenix V|tome|x µCT scanner (General Electric, USA) at the American Museum of Natural History, New York, NY. We segmented crania (407 specimens, 202 species) and mandibles (382 specimens, 191 species) from uCT slices using Mimics v. 19 (Materialise, Leuven, Belgium) and cleaned the resulting models (.STL files) using Geomagic Studio v. 2014 (Geomagic Inc., Research Triangle Park, NC, USA). We digitized three-dimensional landmarks and semi-landmarks from the crania and mandibles (Supplementary Fig. 1 and Supplementary Note 1) of each specimen using Checkpoint v. 2017 (Stratovan, Davis, CA, USA). We oversampled curves in Checkpoint and subsequently resampled them by length using R function digit.curves (package geomorph^54^).

For a small percentage of missing landmarks (chiefly, for specimens with damaged or incomplete zygomatic arches or auditory bullae; 5.5% and 1.7% of all cranial and mandibular landmarks and semi-landmarks, respectively), we used reflected relabeling for those landmarks that could be estimated using a bilaterally symmetrical landmark, and Bayesian Principal Component Analysis (BPCA) to estimate missing values in our datasets (using functions from the R package LOST)^55–57^. Given the low percentage of missing data, the estimation of missing landmarks is unlikely to substantially impact morphospace reconstruction, while also allowing for the inclusion of rare species and more individuals per species^55,56^. We used a simulation approach based on all fully complete specimens to verify that the inclusion of these incomplete specimens was preferred over their exclusion (see Supplementary Note 2).

We applied generalized Procrustes superimposition (GPS) to the cranial and mandibular datasets to remove the effects of scale, rotation and position, using the R function gpagen from the package geomorph^54^, and with the Procrustes distance option for the optimization of semi-landmark positions along curves^58–60^. We calculated the consensus configuration of each species with >1 specimen using the R function mshape (package geomorph). Bilateral landmarks were subsequently averaged after mirroring.

Using phylogenetic principal components analysis (pPCA)^61^, which accounts for the non-independence of trait values due to evolutionary relatedness, we estimated the morphospaces of the cranial and mandibular datasets. We applied pPCA to the covariance matrix of the Procrustes landmark coordinates using the R function phyl.pca from the package phytools^61,62^ using a BM model of evolution due to the size of our datasets. While there has been recent debate in the literature on the issue of phylogenetic correction of PCA^47^, Uyeda et al.^48^ found that standard PCA was associated with considerable bias in model selection when PCA was used to reduce the dimensionality of the dataset prior to macroevolutionary model fitting. This bias was especially strong when shape variation was not concentrated (~70% and higher) on the first PC axis, as is the case with our cranial and mandibular shape datasets. Accordingly, we present the results of the phylogenetically-corrected PCA (pPCA); however, results of the standard PCA and applicable model fitting analyses are included in the supplementary materials (Supplementary Figs. 12–16; Supplementary Tables 4 and 5). We discuss any differences where relevant.

As the sum of the eigenvalues of pPCA are not equal to the variance of the dataset due to transformations from the phylogenetic variance–covariance matrix, we carried out a Procrustes multiple regression of the landmark configuration—after superimposition and averaging—on the scores of the pPCA, using procD.lm from the R package geomorph. We used the *R*^2^ value from the multiple regression to describe the total (absolute) shape variation explained by each axis (see Results and Fig. 1). When calculated using standard PC axes, *R*^2^ values from procD.lm were equivalent to the relative value of the eigenvalues of each axis (Supplementary Fig. 12). We selected a number of critical axes (e.g., those likely representing non-random shape variation) per dataset using parallel analysis with pPCA (or PCA) as a stopping rule^63,64^ (summarized in Supplementary Table 1).

### Morphological disparity across the bat phylogeny and time

To explore how evolutionary processes may have impacted the distribution of modern skull shape diversity in Chiroptera, we employed two methods that allowed for direct comparisons of the full multivariate shape space. Disparity-through-time analysis (DTT) calculates the average morphological disparity of all subclades present in a phylogeny at time *t* compared to the total morphological disparity of all species examined^12^. The area between the observed DTT curves and the median curve from evolutionary simulations is known as the morphological disparity index (MDI)^12–14^. Negative MDI values are indicative that morphological disparity was established early in the history of a clade (e.g., from an early burst of trait evolution). The significance of the observed MDI may be calculated as the percentage of BM simulated curves with a lower MDI^14,65^ (one-sided *p*-value). In contrast with previous studies, which calculate morphological disparity as the average squared pairwise Euclidean distance across a trait or a suite of PC axes^10,13,14,65^, we calculated the mean pairwise Procrustes distances within subclades from the GPS aligned landmarks for the bat cranium and mandible. Thus, we consider disparity in the full 3D shape space.

We followed Zelditch et al.^66^ in using a co-phylogenetic approach to contrast patterns of evolutionary relatedness and morphological diversity. Using the original species (GPS aligned) landmarks, pairwise Procrustes distances were calculated among all species pairs. This was input as a dissimilarity matrix in a hierarchical clustering analysis using UPGMA (R function hclust). We compared the configuration of the phylogeny and dendrogram of cranial and mandibular morphology using a tanglegram produced by the R function cophylo (package phytools^62^), which rotates nodes in the phylogeny and dendrogram to optimize tip matching (see Fig. 3). Similarity between morphological and phylogenetic relationships in tanglegrams are indicated by ~ parallel lines linking the same species in the phylogeny and the morphology dendrogram. Comparatively, mismatches between morphology and evolutionary relatedness are indicated by steep connecting lines in a tanglegram, and may suggest convergent taxa or phenotypic innovations^66^.

We quantified phylogeny–morphology discordance and contrasted it with expectations from a random-walk evolutionary model (BM)^67^. For our observed bat cranial and mandibular tanglegrams, we calculated a tree-wide metric of tip displacement as the average of the counts of vertical displacements for each species in the phylogeny (i.e., for a species located in the same position in both trees, tip displacement = 0). We additionally simulated 1000 landmark datasets using functions sim.char and rate.matrix from the R package geiger^65^, and calculated the average of all tip displacements for each simulated tanglegram (Supplementary Fig. 9). We determined the significance (one-sided *p*-value) of the observed tip displacement as the percentage of simulated values lower (i.e., greater match between phylogeny and morphology) than that observed across cranial and mandible shape. We illustrate the appearance of simulated tanglegrams, which produced tip displacement values near the median of all simulations, in Supplementary Fig. 5.

### Skull shape adaptive landscape

Maximum likelihood model fitting approaches have been widely used to quantify and compare evolutionary patterns in continuous morphological traits (e.g., linear morphometrics, geometric morphometrics, functional morphology, biomechanical indices, etc.^11,21,67–69^). Evolutionary models may represent trait evolution under a random walk process under a constant rate (BM), or variable rate (e.g., Early Bursts, EB). These models may further incorporate selective constraint, such as Ornstein–Uhlenbeck processes, which additionally include an elastic selection parameter^70,71^ that pulls trait evolution towards one or several optimum values. Recently developed methods have applied these multi-peak OU models to detect shifts in the pattern of morphological evolution^22,38,44^, creating an adaptive landscape of trait values that reflects Simpsons’s framework of “adaptive zones” across multivariate trait spaces and multiple selective optima. These last approaches are particularly powerful in that they do not require an a priori hypothesis of shift locations^22^ (e.g., with changes in ecology or environments), and can be contrasted with specific hypotheses regarding trait evolution (changing rates, impacts of innovations, ecological adaptations, etc.)

We applied l1ou^38^ to multivariate morphospaces (the scores of all critical axes, see geometric morphometric methods above) representing cranial and mandibular shape diversity across bats to estimate the configuration of adaptive shifts. The l1ou approach uses a lasso method to estimate the coefficients in a sparse vector of potential adaptive regime shift positions (i.e., phylogenetic branches), is more computationally efficient than some other methods^22^, and is able to accommodate multiple trait axes^44^. We note that while evolutionary shifts are contrasted across multiple trait axes simultaneously, l1ou does not incorporate trait covariances.

We applied l1ou to cranial and mandibular principal component scores (both pPCA and PCA) across all sampled species (using the Shi and Rabosky phylogeny^23^) as well as within the Phyllostomidae alone (using the Rojas et al. phylogeny^51^). For computational efficiency, we limited the number of potential shifts to 50 and 20 for the full dataset and phyllostomid dataset respectively. We selected the best fit shift configurations using pBIC, a modification of BIC that accounts for phylogenetic correlation among shifts^38^ and is more conservative than Akaike Information Criterion (AIC) or Bayesian Information Criteria (BIC) alone. We scaled PC scores by 100 to avoid computational issues of estimating evolutionary parameters very close to zero (this only changes the scaling of the parameters, and not the differences in model fit).

### Comparisons with diet and echolocation

We contrasted the adaptive landscape model generated by l1ou with other potential drivers of skull shape evolution in bats, including models of rate heterogeneity (i.e., an early burst predicted under an adaptive radiation) and models reflecting ecological or performance traits thought to constrain bat skull evolution (e.g., dietary ecology and echolocation). Fully multivariate evolutionary model fitting was implemented using mvMORPH^72^. While l1ou ignores trait covariance while estimating adaptive shift positions, mvMORPH includes all trait covariances in calculating model support. Using mvMORPH, we fit multivariate models for several constant rate or rate-change evolutionary models (a single-rate BM process, a single, global OU process, and an EB model that allows for the slowing of evolutionary rates through time) to the standard and phylogenetically-corrected morphospaces (PCA and pPCA scores, respectively). We also fit multi-peak OU models representing shift configurations for the l1ou models as well as the reconstructions of diet and echolocation (see below). Models were contrasted using sample-size corrected Akaike Information Criterion (AICc). We note that effect size was not calculated for multi-peak OU models, as these methods are available only for univariate OU-models, and we evaluate only the relative fit of the models.

Echolocation type was categorized as three states based on the presence of laryngeal echolocation and emission of calls (non-laryngeal echolocators, oral-emitting echolocators and nasal-emitting echolocators) and using assessments from prior literature^25–27,73^. Species were assigned echolocation type based on the primary mode of echolocation. However, we must note that patterns of nasal and oral emission are more complex than represented by this discrete character. At least some species from primarily oral-emitting clades (especially vespertillionids within *Corynorhinus*, *Plecotus*, and *Barbastella*) may facultatively employ nasal-emission, and some nasal-echolocating species within Phyllostomidae have been observed to fly with an open mouth (Fenton, pers. comm.), potentially incorporating both nasal and oral emission^41,74–76^. All pteropodids have lost laryngeal echolocation, although it is worth noting that at least one genus (*Rousettus*) echolocates through tongue-clicks^77^.

Dietary categories included frugivores, nectarivores, insectivores, carnivores (significant consumption of vertebrates), omnivores (significant consumption of both animal and plant resources) and sanguivores (blood-feeding), based on previous diet assessments^27,28,30,34^. We note that dietary variation in bats is more complex than represented by these seven states alone (e.g., adaptations such as durophagy in species such as *Centurio senex* and piscivory in *Noctilio leporinus*), however dietary diversity (in dietary breadth, functional properties, seasonal variation, etc.) is poorly documented for most bat species.

Diet and echolocation type were reconstructed across the phylogeny using stochastic character mapping SIMMAP^78,79^ as implemented in the R phytools function make.simmap (100 simulations per tree; Supplementary Figs. 6 and 8). Shift configurations determined by l1ou for the cranium and mandible were converted into a simmap format using paintSubTree in R package phytools^62^.

### Reporting summary

Further information on research design is available in the Nature Research Reporting Summary linked to this article.

## Supplementary information

Supplementary Information

Reporting Summary

Source Data

## Data Availability

Phylogenies are available through their respective publications^26,54^. Source data files are available for raw geometric morphometric data, specimen information, all pPCA and PCA figures, DTT plots, and missing data analyses (Figs. 1, 2, 6; Supplementary Figs. 2–3, 6–12, 15–16).

## Code availability

All major R functions used in this manuscript are taken from the previously published packages, geiger, phytools, geomorph, l1ou, mvMORPH, LOST, and the base stats package. Specific functions are provided with the relevant analyses in the Methods section.
