# Supplementary material for: Signatures of echolocation and dietary ecology in the adaptive evolution of skull shape in bats
Source: Nat Commun. 2019 May 2;10:2036. doi: 10.1038/s41467-019-09951-y (PMC6497661; doi:10.1038/s41467-019-09951-y)
Supplement: Supplementary file 1 — Supplementary Information [file 41467_2019_9951_MOESM1_ESM.pdf]

SUPPLEMENTARY INFORMATION

Signatures of echolocation and dietary ecology in the adaptive evolution of skull shape in bats

Arbour et al.

SUPPLEMENTARY NOTE 1: GEOMETRIC MORPHOMETRIC METHODS

Description of Landmarks

Mandible Landmarks
1. Anteriormost point on the mandibular symphysis
2-3. Anteriormost point on canine alveolus
4-5. Anteriormost point on premolar alveolus
6-7. Anteriormost point of first molar alveolus
8-9. Lateralmost point on mandibular condyle
10-11. Medialmost point on mandibular condyle
12-13. Inflection point on the posterior profile between the mandibular condyle and the angular process
14. Ventralmost point on the mandibular symphysis
15-16. Posteriormost point on the angular process
17-18. Posteriormost point on the last molar alveolus
19-20. Anterior inflection point on the articular surface, midpoint between L8/9 and L10/11.

Curves with equidistant sliding semi-landmarks
1-2, From L17/18 to L19/20, along the dorsal profile of the coronoid process. (blue)
3-4, From L14 to L15-16, along the ventral profile of the ramus and angular process. (red)

Cranial Landmarks
1-2. Anteriormost point of premaxilla
3-4. Anteriormost point on canine alveolus
5-6. Anteriormost point on premolar alveolus
7-8. Anteriomost point on the first molar alveolus
9-10. Posteriormost point on the last molar alveolus
11-12. Ventralmost point on pterygoid hamulus
13-14. Medialmost margin of the mandibular fossa
15-16. Ventralmost point on mastoid process
17-18. Dorsalmost point on the external edge of the auditory meatus
19-20. Ventralmost point on the external edge of the auditory meatus
21-22. Lateralmost point on occipital condyle
23. Dorsal border of foramen magnum
24. Ventral border of foramen magnum
25. Posteriormost point on midline of palate
26. Anteriormost point on midline of the complete palate
27. Anteriormost point on the midline of the nasals
28 Posteriormost point on the intersection of the lambdoidal and sagittal crests
29-30. Anteriormost point on the inflection of the orbit
31-32. Posteriormost point on the intersection of the zygomatic arch and braincase
33-34. Ventralmost point of the insertion of the zygomatic arch on the maxilla
35-36 Lateralmost point on the margin of the mandibular fossa

Curves with equidistance sliding semi-landmarks:
1. From L27 to L28, along the dorsal midline of the cranium (blue)
2-3. From L29/30 to L31/32, along the dorsal profile of the zygomatic arch (red)
4-5. From L33/34 to L35/36, along the ventral profile of the zygomatic arch (yellow)

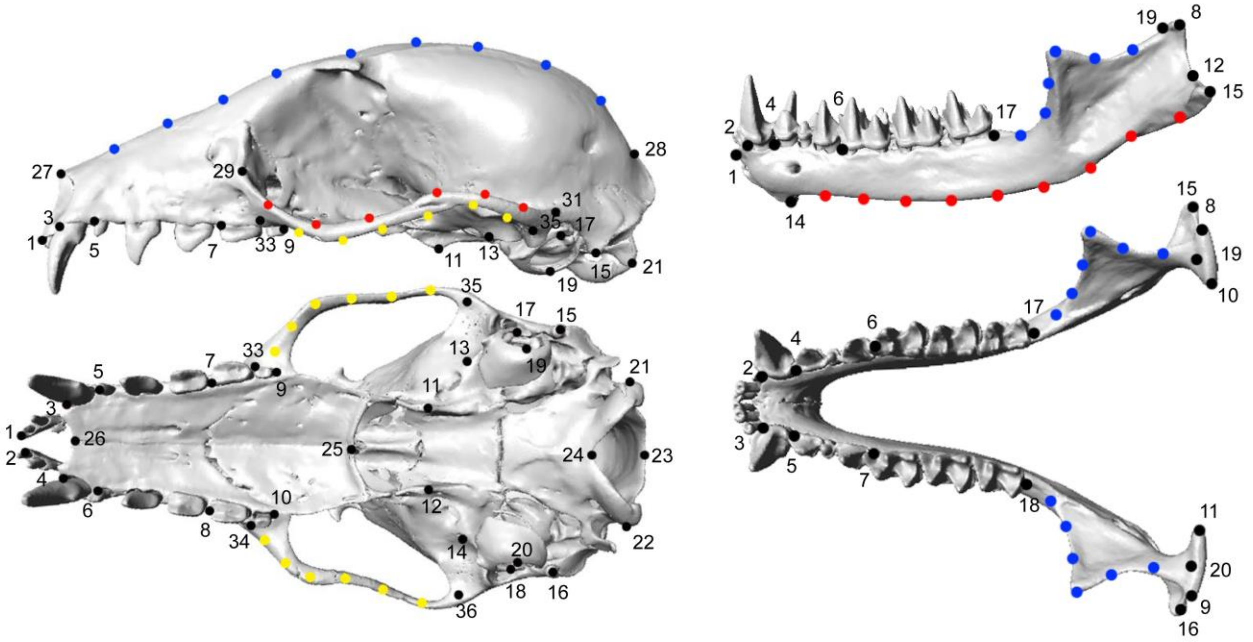

Supplementary Figure 1: Location of 3D geometric morphometric landmarks on the cranium and mandible of *Eonycteris spelaea*. Landmarks are given by black points, semi-landmarks by coloured points (see landmark list above).

Supplementary Table 1: Number of critical principal components per anatomical structure (cranium or mandible) and per clade (all bats or phyllostomids), with (pPCA) or without (PCA) phylogenetic correction. PC scores from these axes were used in all subsequent macroevolutionary model fitting analyses.

	**Number of Species**	**pPCA axes**	**PCA axes**
**All bats**			
**cranium**	202	3	4
**mandible**	191	4	4
**Phyllostomidae**			
**cranium**	38	3	3
**mandible**	38	2	3

SUPPLEMENTARY NOTE 2: IMPACT OF THE ESTIMATION OF MISSING DATA

Methods:

Geometric morphometric methods are currently intolerant of missing data. We follow the approach of Arbour and Brown[1] to evaluate the effect of missing landmark coordinate data and specimen incompleteness on the resulting morphospaces of the cranium and mandible. Namely, we evaluated whether it was better to estimate data missing from incomplete specimens, or to exclude those specimens entirely. While both datasets have a low overall proportion of missing data, this is spread across a large number of specimens and landmarks (e.g., ~40% of crania and 78% of cranial landmarks have at least 1 missing value). Therefore, the exclusion of missing data would substantially limit our dataset, especially for rare taxa with lower sample sizes. To test for the impact of missing data estimation, we used the following steps (and illustrated in workflow below).

1) We selected from our data a subset representing all specimens with complete landmark sets as our training dataset (dataset COM). We assume that this dataset better reflects the variance associated with landmark positions across bats than either datasets with missing data estimated, or incomplete specimens excluded.
2) From a chosen proportion of specimens, we randomly sampled landmarks based on the distribution of missing landmarks from our real incomplete specimens, and removed the corresponding coordinate data. Missing values were subsequently estimated using either "reflected relabeling" or BPCA (see below), and this formed the dataset EST.
3) We then excluded all simulated incomplete specimens (dataset EXC).
4) We evaluated the change in the variance structure of the dataset imparted by estimation or exclusion. We used the R function "procrustes" (from the package "vegan") to quantify the fit between the eigenvectors of COM vs. EST and COM vs. EXC as calculated by the "Procrustes sum of squares" (PSS). Lower values of PSS indicate a stronger fit between datasets.

For step 2, we first exploited bilateral symmetry across the cranium and mandible. Missing bilaterally symmetrical landmarks can be estimated using "reflected relabeling"[2], wherein the specimen is mirrored and aligned with the (overlapping) original landmarks using Procrustes superimposition; missing landmarks on either side are then imputed from the mirrored specimen. We applied reflected relabeling to the landmark data using the R function "flipped" from the package LOST[3]. For those missing landmarks that could not be estimated using reflected relabeling (3.2% and 0.4% of all specimen cranial and mandibular landmarks and semi-landmarks, respectively), we employed a Bayesian Principal Component Analysis approach[4], using the R function "MissingGeoMorph" from the package "LOST" [1,3,5].

The preceding four steps were carried out on each of the cranium and mandible datasets across a range of values for the proportion of incomplete specimens, based on that observed in each of our datasets (2% to 20% of mandibles and 30 to 50% of all crania). We simulated 500 datasets with missing data per value of the proportion of incomplete specimens. This helps to prevent the evaluation of axes with eigenvalues lower than from random datasets, and less likely to represent useful shape variation. We also carried out PCA both with (pPCA) and without (PCA) phylogenetic correction. To improve comparability between PSS values across PCA and estimation methods (and because the EXC datasets contains fewer eigenvectors than the COM or EST datasets), we used the eigenvectors for the number of critical axes observed in the main results (3 PC axes from the cranium, 4 PC axes from the mandible).

Additionally, we tested the relative impact of estimating missing data by exploiting bilateral symmetry from each individual specimen, or by comparisons with the full dataset. For each of the cranium and mandible we created two datasets with missing landmarks, a) one with landmarks missing from only one side (to evaluate reflected relabeling, see methods) and b) one with landmarks missing from either side (to evaluate BPCA, see methods). We selected BPCA for non-paired missing landmarks as it has shown to reliably estimate missing data from a variety of multidimensional morphometric datasets, across a range of sample sizes and taxonomic scopes [1,5].

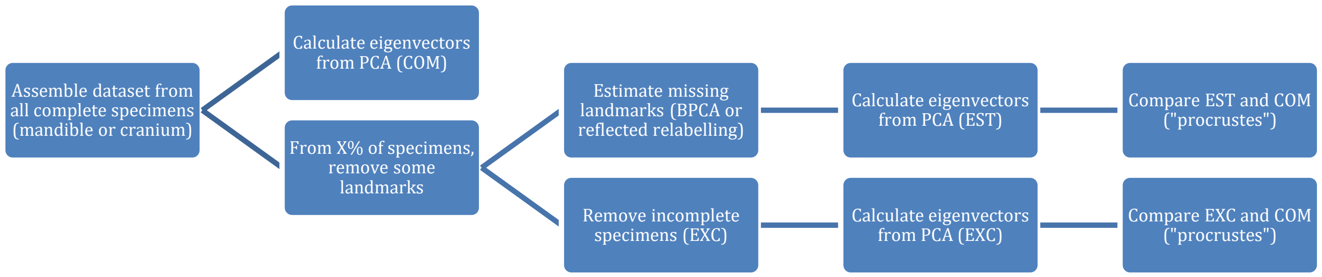

<u>Results:</u>

Across both the cranium and mandible, the impact of missing data estimation was less than the impact of excluding incomplete specimens at approximating the shape variance associated with all fully complete specimens. At low proportions of missing specimens, the PSS generated by the EST and EXC mandible datasets overlapped (Supplementary Figure 3), however at 2% incomplete mandibles (which showed the greatest overlap) the number of individual datasets for which the PSS of EXC was lower than the PSS of EST was only 0.2%. Additionally, we would expect the results of EXC and EST to converge as the proportion of incomplete specimens decreases, as both datasets contain almost all specimens present in COM. The median value of PSS using BPCA was consistently lower than the EXC dataset for the cranial dataset. However, the absolute range of PSS also overlapped when missing data in the cranial dataset was estimated using BPCA. Similar to the mandible dataset, across most individual datasets estimation was preferred over exclusion (e.g., at 40% incomplete specimens, only 2.8% of simulated datasets showed lower PSS values for EXC than EST, and at 30% incomplete specimens, only 3.8% of simulated datasets showed lower PSS values for EXC than EST).

"Reflected relabeling" performed better than BPCA across both datasets, likely as a result of better incorporating intra-individual and intra-specific shape variation. A large proportion of missing data in our original datasets represented landmarks that could be estimated by exploiting bilateral symmetry (see main text methods), and our initial selection of damaged skulls was generally biased towards such specimens. Thus, our analysis of the impact of BPCA is conservative, as markedly fewer landmarks needed to be estimated this way than represented in our simulated datasets.

These results are consistent with previous analyses of the impact of incomplete specimens in geometric morphometric analyses, which showed that for most estimation approaches it was generally better to estimate missing data than exclude incomplete specimens.

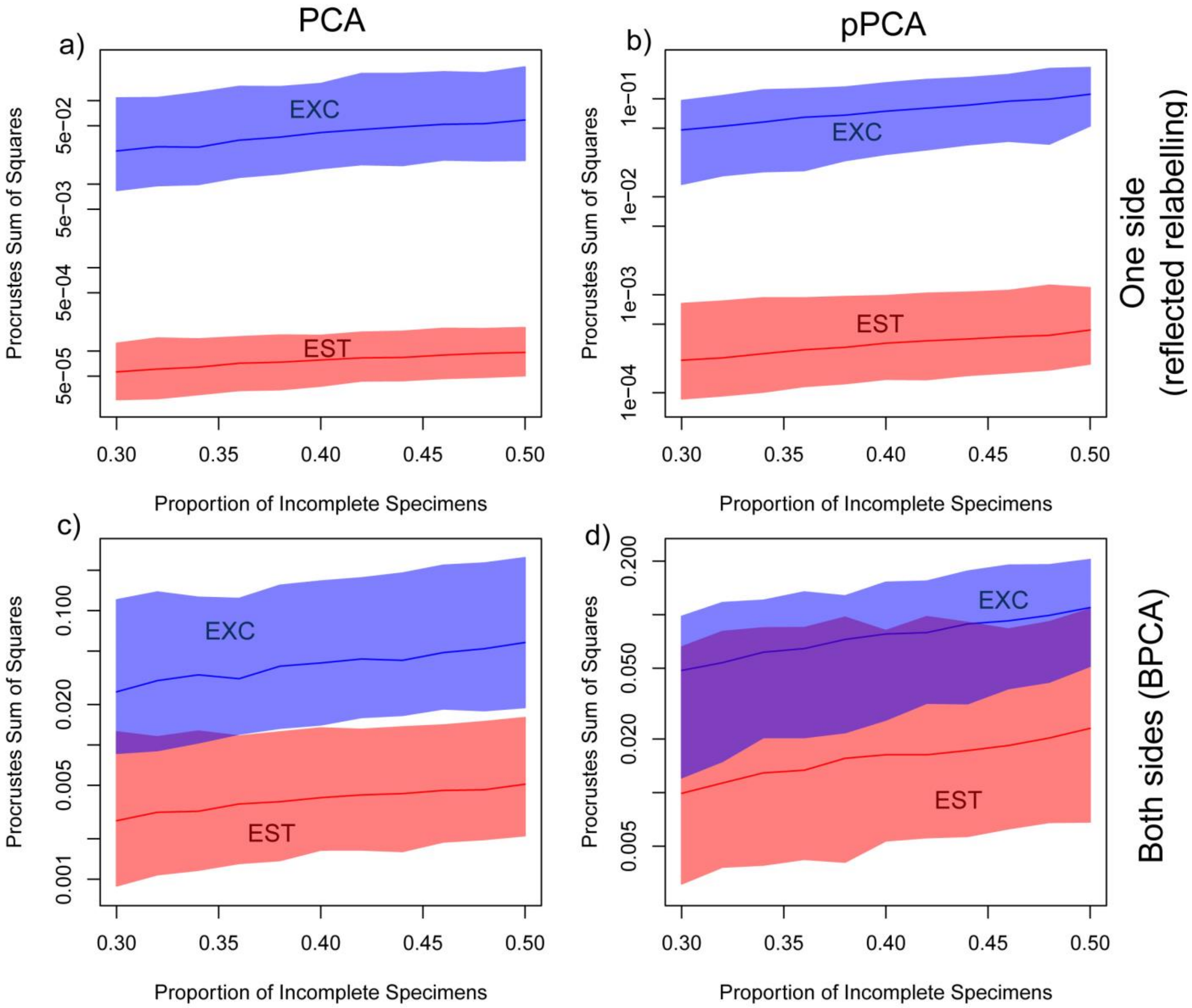

Supplementary Figure 2: Results of missing data analyses of cranium morphology. The fit between the eigenvectors, of either PCA (a and c) or pPCA (b and d), of the COM dataset (complete specimens) versus either the EST dataset (missing data estimated) or the EXC dataset (incomplete specimens excluded) were determined using Procrustes-sum-of-squares (PSS) from the R function "procrustes". Missing data was estimated using reflected relabeling (a-b) or BPCA (c-d). Lines indicate the median and shaded area indicate the 95% range of 500 simulated incomplete datasets. See source data file.

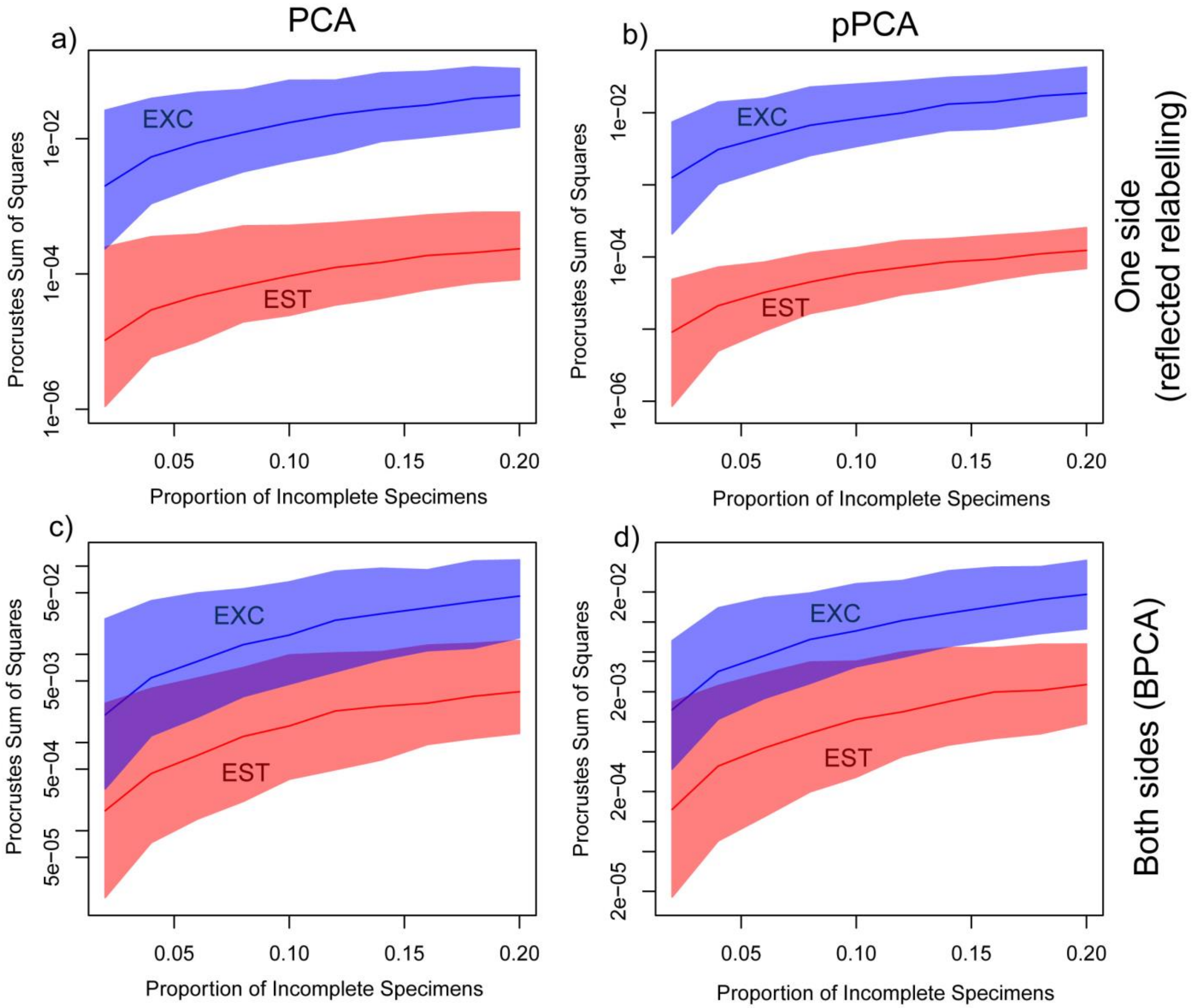

Supplementary Figure 3: Results of missing data analyses of mandible morphology. The fit between the eigenvectors, of either PCA (a and c) or pPCA (b and d), of the COM dataset (complete specimens) versus either the EST dataset (missing data estimated) or the EXC dataset (incomplete specimens excluded) were determined using Procrustes-sum-of-squares (PSS) from the R function "procrustes". Missing data was estimated using reflected relabeling (a-b) or BPCA (c-d). Lines indicate the median and shaded area indicate the 95% range of 500 simulated incomplete datasets. See source data file.

SUPPLEMENTARY FIGURES

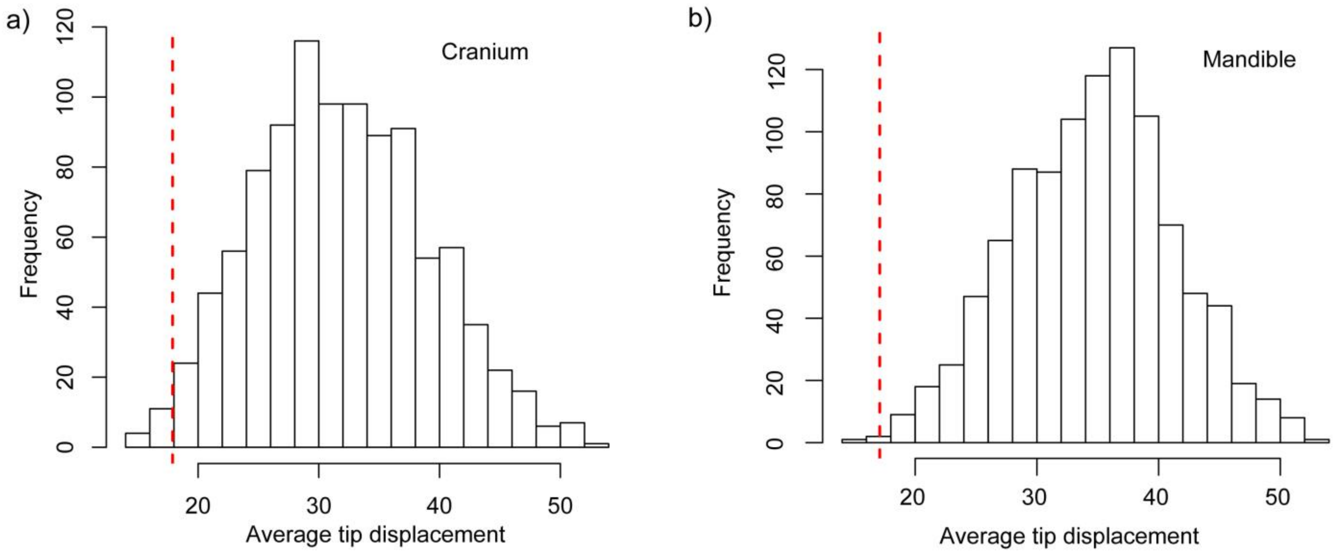

Supplementary Figure 4: Distribution of tip displacement values from 1000 Brownian Motion simulations of character history for the cranium and mandible of bats (and see methods). Dashed line gives the observed values for the cranium (a) and mandible (b).

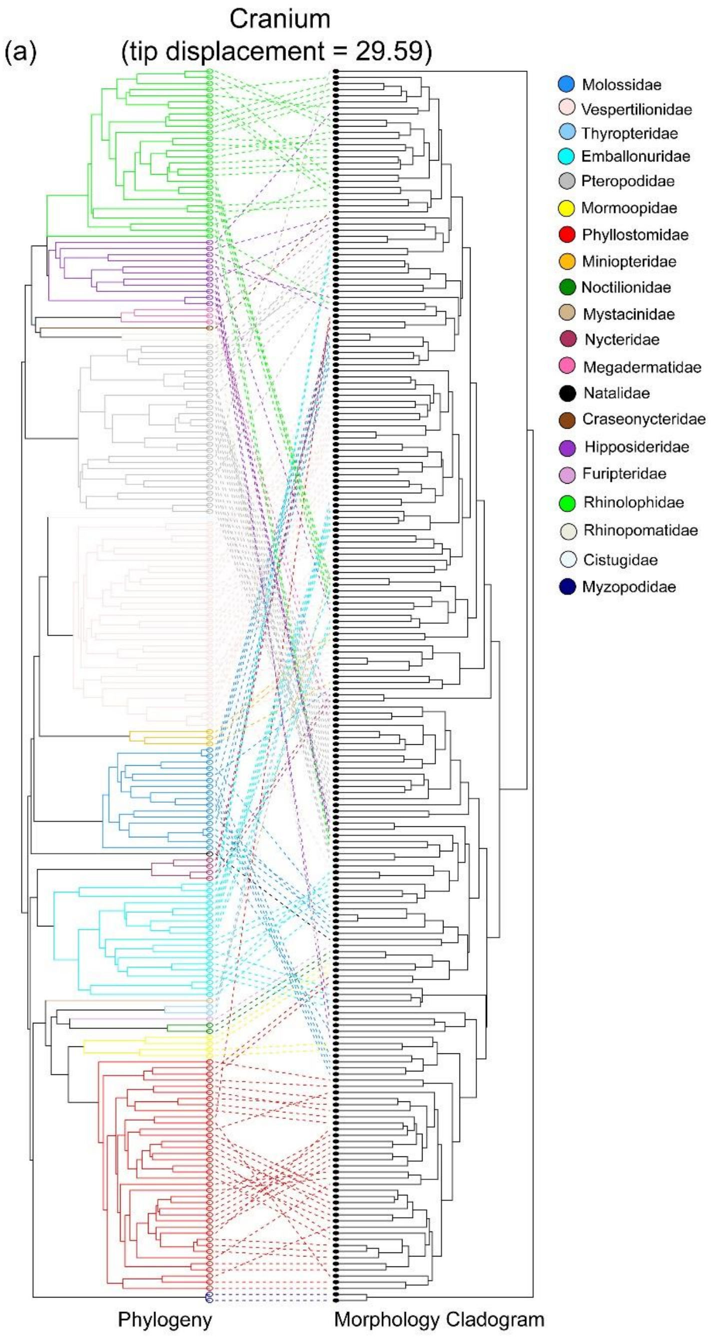

Supplementary Figure 5: Tanglegrams of phylogenetic relationships of Chiroptera (left) and dendrograms from BM simulated morphological traits. Simulated character datasets for the cranium (a) and mandible (b) were selected to have a tip displacement value near the median of 1000 BM simulations (and see Supplementary Figure 4). Dotted lines link the same species in both trees.

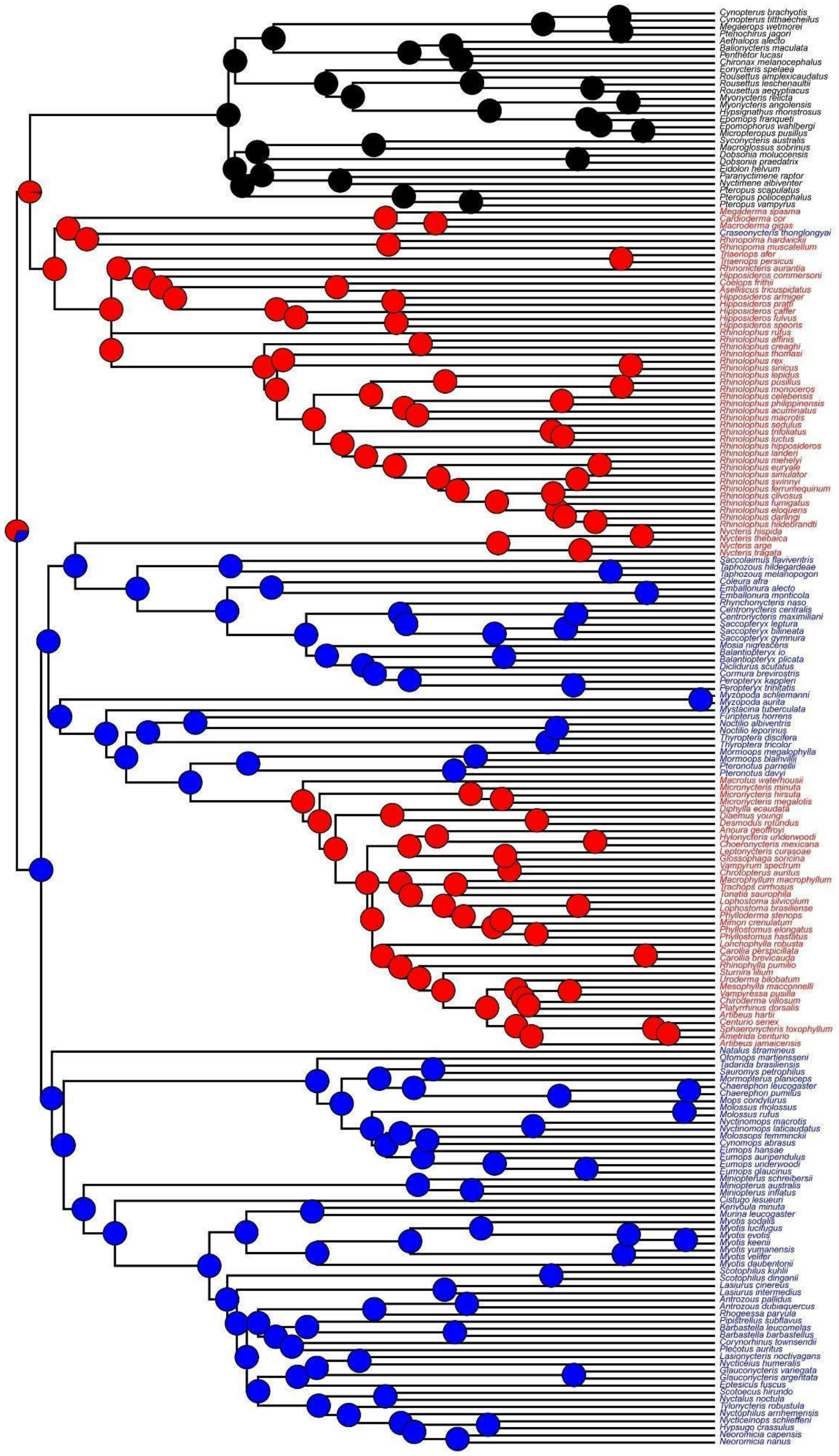

Supplementary Figure 6: Reconstruction of echolocation type across 202 species of bat. Black = non-laryngeal echolocators, Blue = Oral-emitting laryngeal echolocators, Red = nasal-emitting laryngeal echolocators. Based on 100 SIMMAP character reconstruction, pie charts show posterior support per node. See source data file.

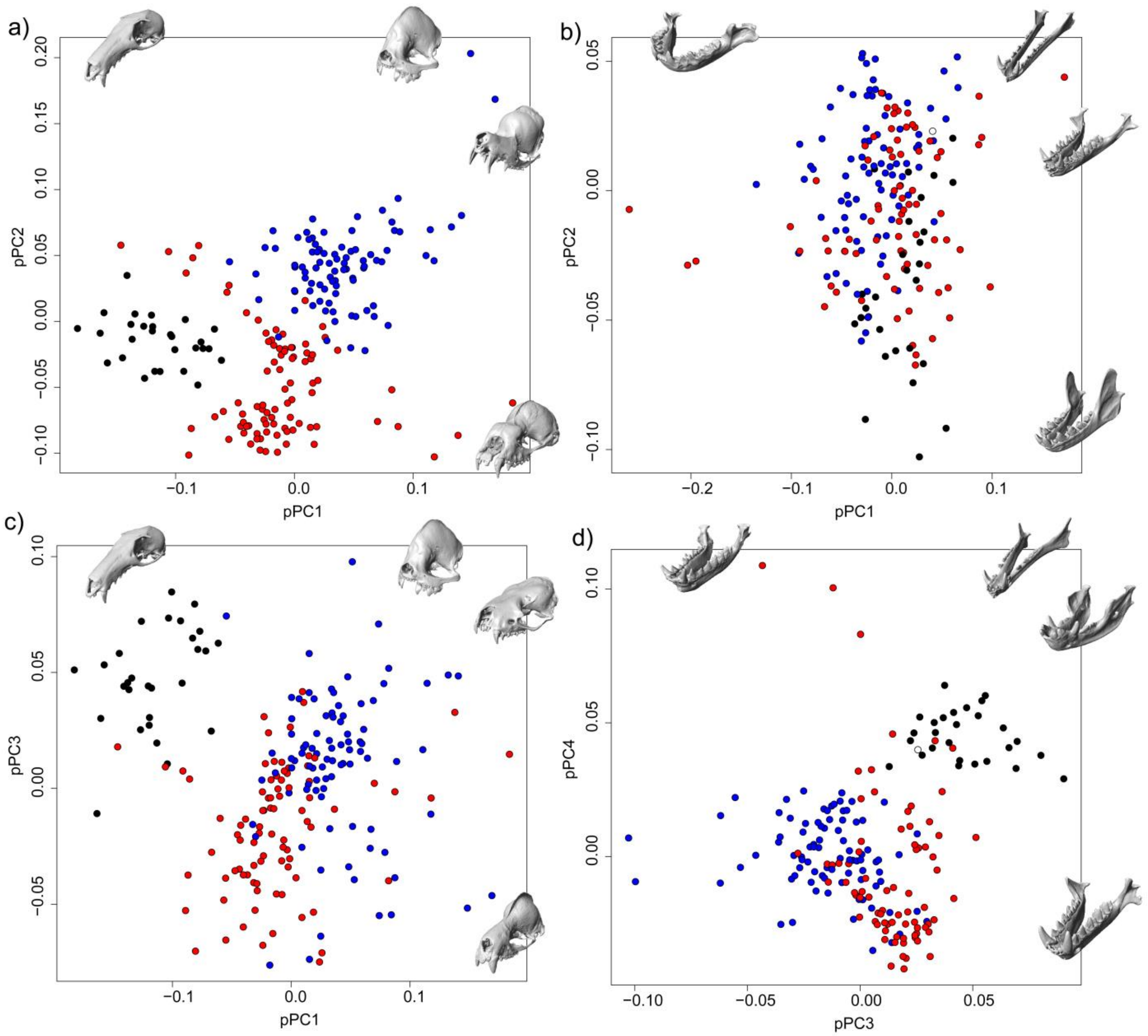

Supplementary Figure 7: Echolocation type of bats in cranial and mandibular morphospace (and see Fig. 1 and Fig. 2). Scores for pPC 1-3 for the cranium (a and c) and the mandible (b and d), coloured by echolocation type. Black = Non-laryngeal echolocators, Red = Nasal-emitting laryngeal echolocators, Blue = Oral-emitting laryngeal echolocators. See source data file.

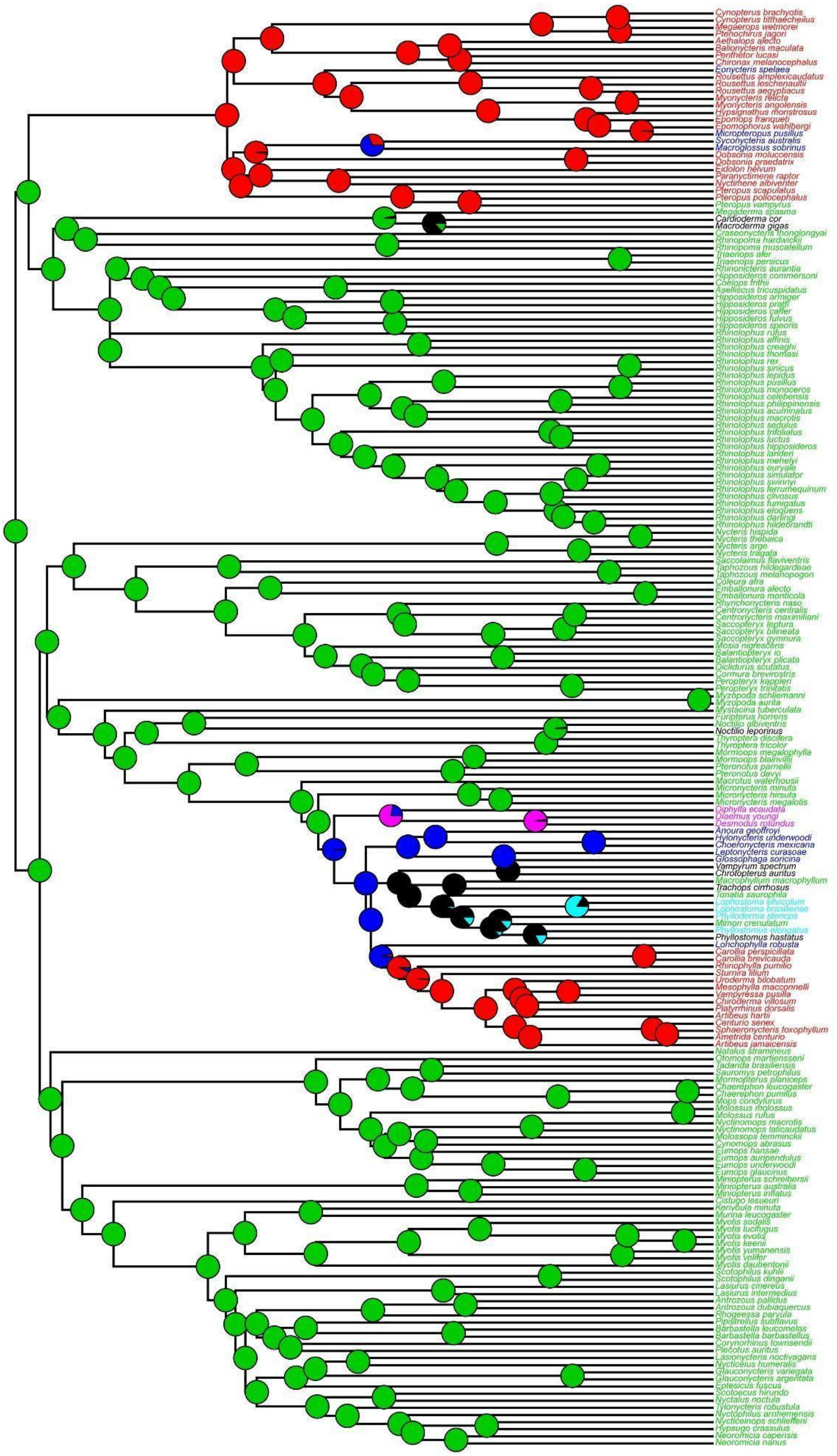

Supplementary Figure 8: Reconstruction of dietary niche across 202 species of bat. Red = frugivore, Blue = nectarivore, Black = carnivore, Green = insectivore, Cyan = Omnivore, Magenta = Sanguivore. Based on 100 SIMMAP character reconstruction, pie charts show posterior support per node. See source data file.

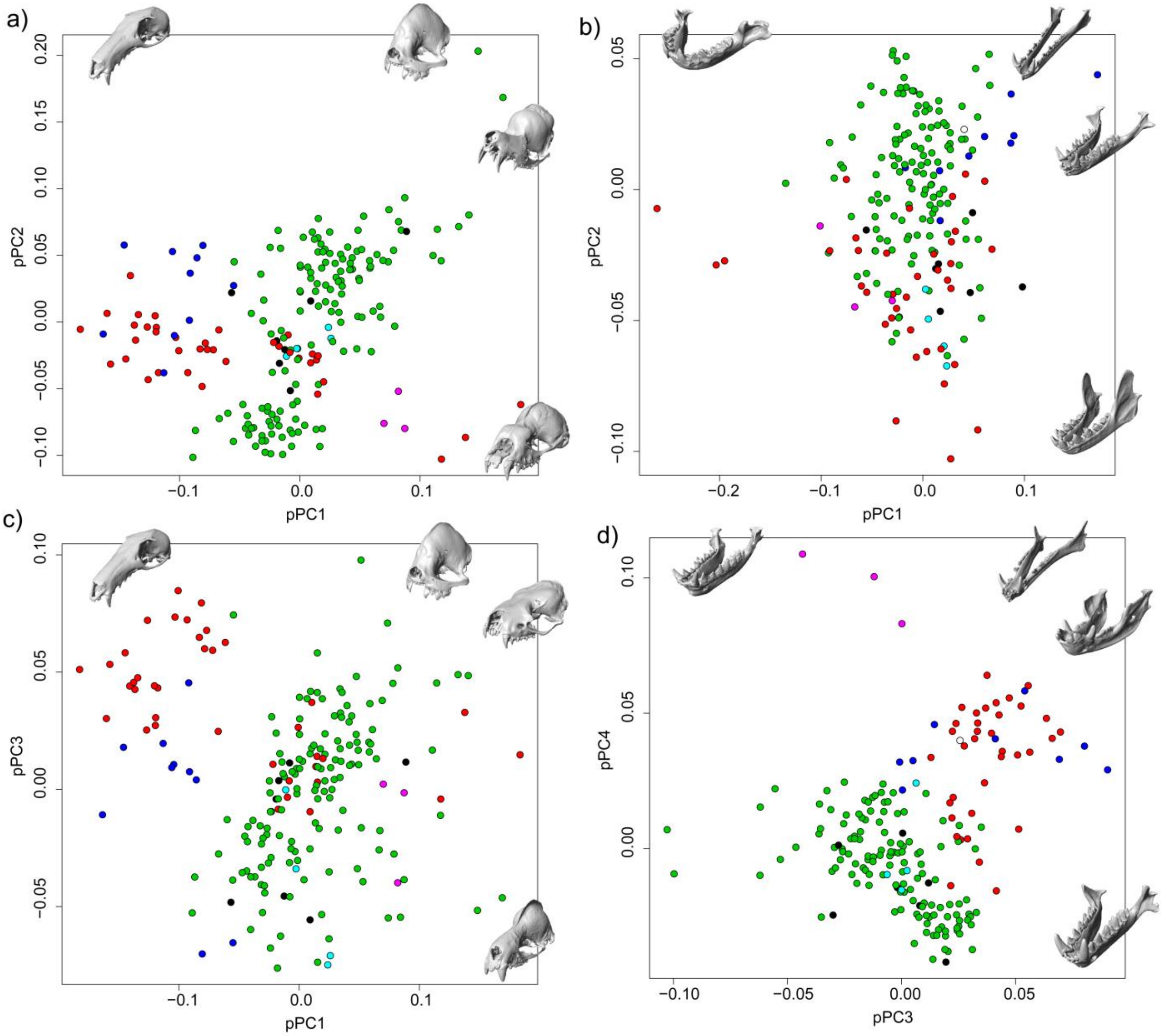

Supplementary Figure 9: Dietary niche in cranial and mandibular morphospace (see Fig. 1). Scores for pPC 1-3 for the cranium (a and c) and the mandible (b and d), coloured by diet. Red = Frugivore, Blue = Nectarivore, Green = Insectivore, Black = Carnivore, Magenta = Sanguivore, Cyan = Omnivore. See source data file.

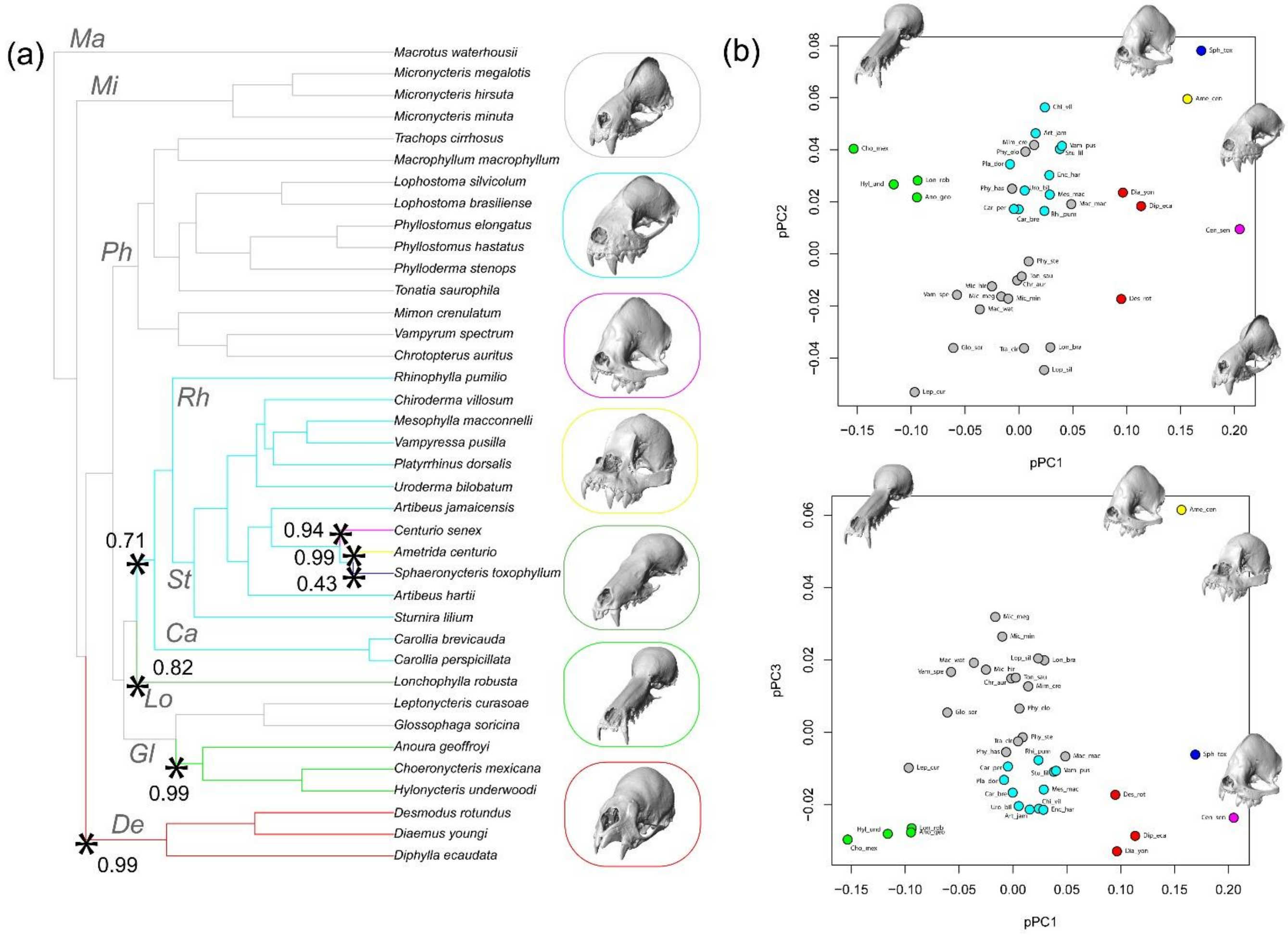

Supplementary Figure 10: Evolutionary shifts (*) in cranium shape in phyllostomids. A) Location of adaptive shifts, as determined by “l1ou” adaptive landscape model fitting on pPCA scores. Bootstrap support given for shift locations. Representative taxa of well supported shifts, from top to bottom: *Lophostoma silvicolum, Artibeus jamaicensis, Centurio senex, Ametrida centurio, Lonchophylla robusta, Choeronycteris mexicana, Desmodus rotundus.* Subfamilies in grey text: Ma = Macrotinae, Mi = Micronycterinae, Ph = Phyllostominae, Rh = Rhinophyllinae, St = Stenodermatinae, Ca = Carollinae, Lo = Lonchophyllinae, Gl = Glossophaginae, De = Desmodontinae. B) Cranial morphospace of phyllostomid bats based on phylogenetic PCA. Taxa illustrated: pPC1 (+ *Centurio senex*, - *Choeronycteris mexicana*), pPC2 (+ *Artibeus jamaicensis*, - *Lophostoma silvicolum*), pPC3 (+ *Ametrida centurio*, - *Centurio senex*). See source data file.

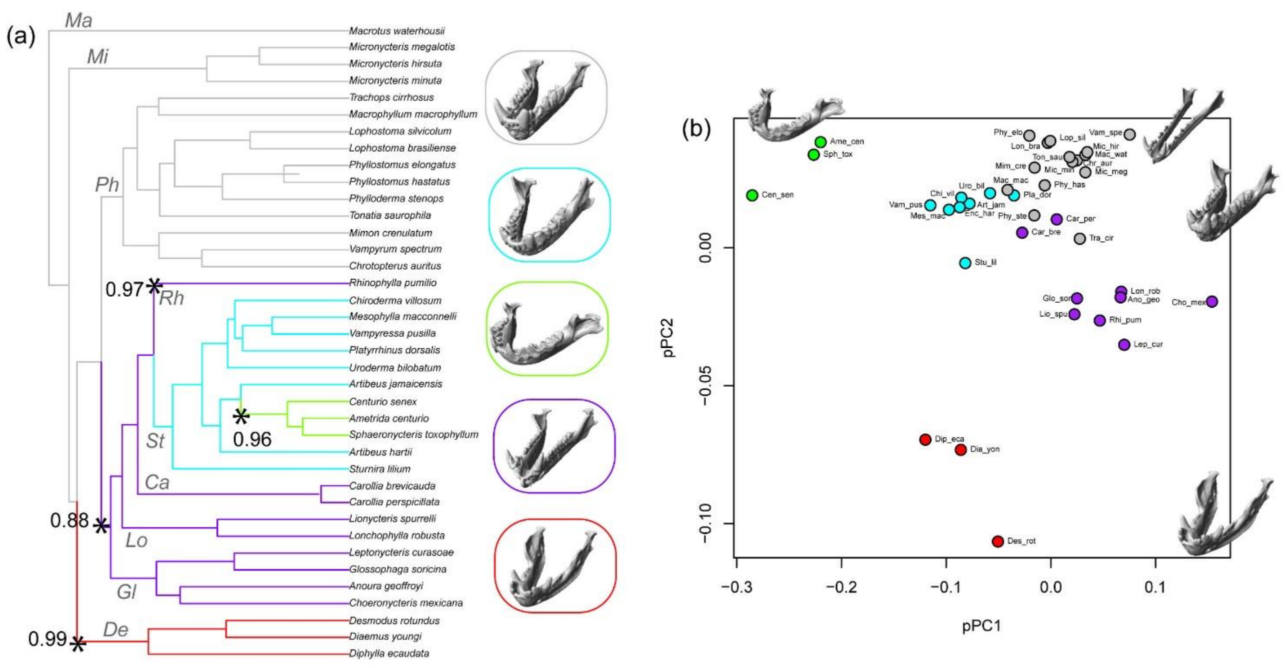

Supplementary Figure 11: Evolutionary shifts (*) in mandible shape in phyllostomids. A) Location of adaptive shifts, as determined by "l1ou" adaptive landscape model fitting on pPCA scores. Bootstrap support given for shift locations. Representative taxa of well supported shifts, from top to bottom: *Trachops cirrhosus*, *Centurio senex*, *Sturnira lilium*, *Anoura geoffroyi*, *Desmodus rotundus*. Subfamilies as in Supplementary Figure 10. B) Mandibular morphospace of phyllostomid bats based on phylogenetic PCA. Taxa illustrated: pPC1 (- *Centurio senex*, + *Choeronycteris mexicana*), pPC2 (+ *Micronycteris hirsuta*, - *Desmodus rotundus*). See source data file.

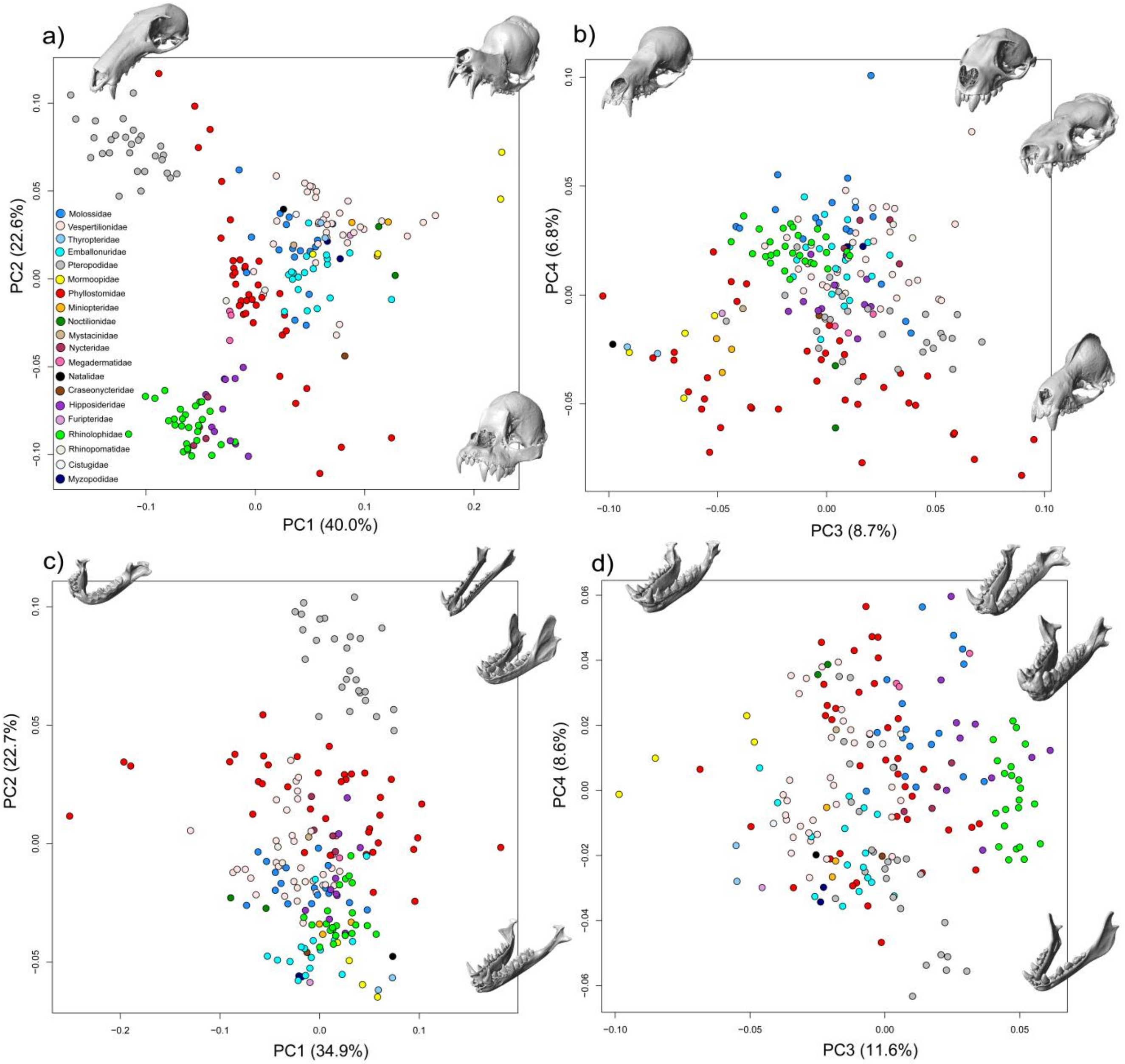

Supplementary Figure 12: Cranial and mandibular morphospaces of bats based on PCA scores. Taxa illustrated: Cranium (a-b) – PC1 (- *Macroglossus sobrinus*, + *Mormoops blainvillei*), PC2 (- *Ametrida centurio*, + *Mormoops blainvillei*), PC3 (- *Leptonycteris curasoae*, + *Nyctimene albiventer*), PC4 (+ *Sauromys petrophilus*, *Lophostoma silvicolum*); Mandible (c-d) – PC1 (+ *Choeronycteris mexicanus*, - *Centurio senex*), PC2 (+ *Myzopoda aurita*, + *Dobsonia praedatrix*), PC3 (+ *Rhinolophus ferrumequinum*, - *Mormoops megalophylla*), PC4 (+ *Micronycteris hirsuta*, - *Micropteropus pusillus*). Percent value gives the shape variation associated with each axis. See source data file.

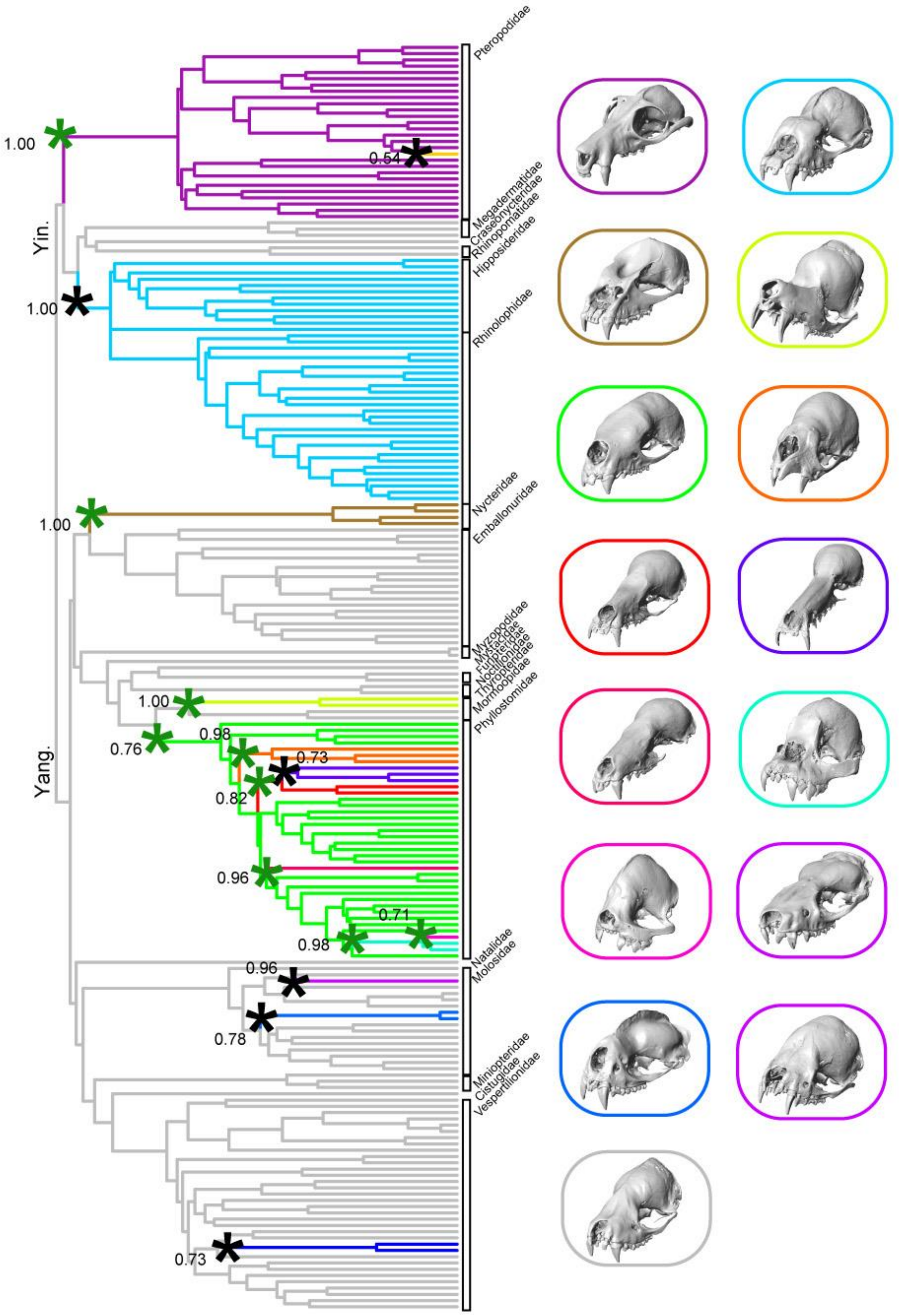

Supplementary Figure 13: Evolutionary shifts (*) in cranium shape across bats, as determined by "l1ou" adaptive landscape model fitting on PCA scores (PC 1-4; Supplementary Figure 12). Bootstrap support given for shift locations. Green shifts = also found in analyses of pPCA scores. Representative taxa, from top to bottom, Left column: Pteropus poliocephalus, Nycteris hispida, Phylloderma stenops, Leptonycteris curasoae, Lonchophylla robusta, Centurio senex, Molossus rufus, Murina leucogaster; Right column: Hipposideros caffer, Mormoops blainvillei, Desmodus rotundus, Choeronycteris mexicanus, Ametrida centurio, *Sauromys petrophilus*. Yin. = Yinpterochiroptera. Yang. = Yangochiroptera.

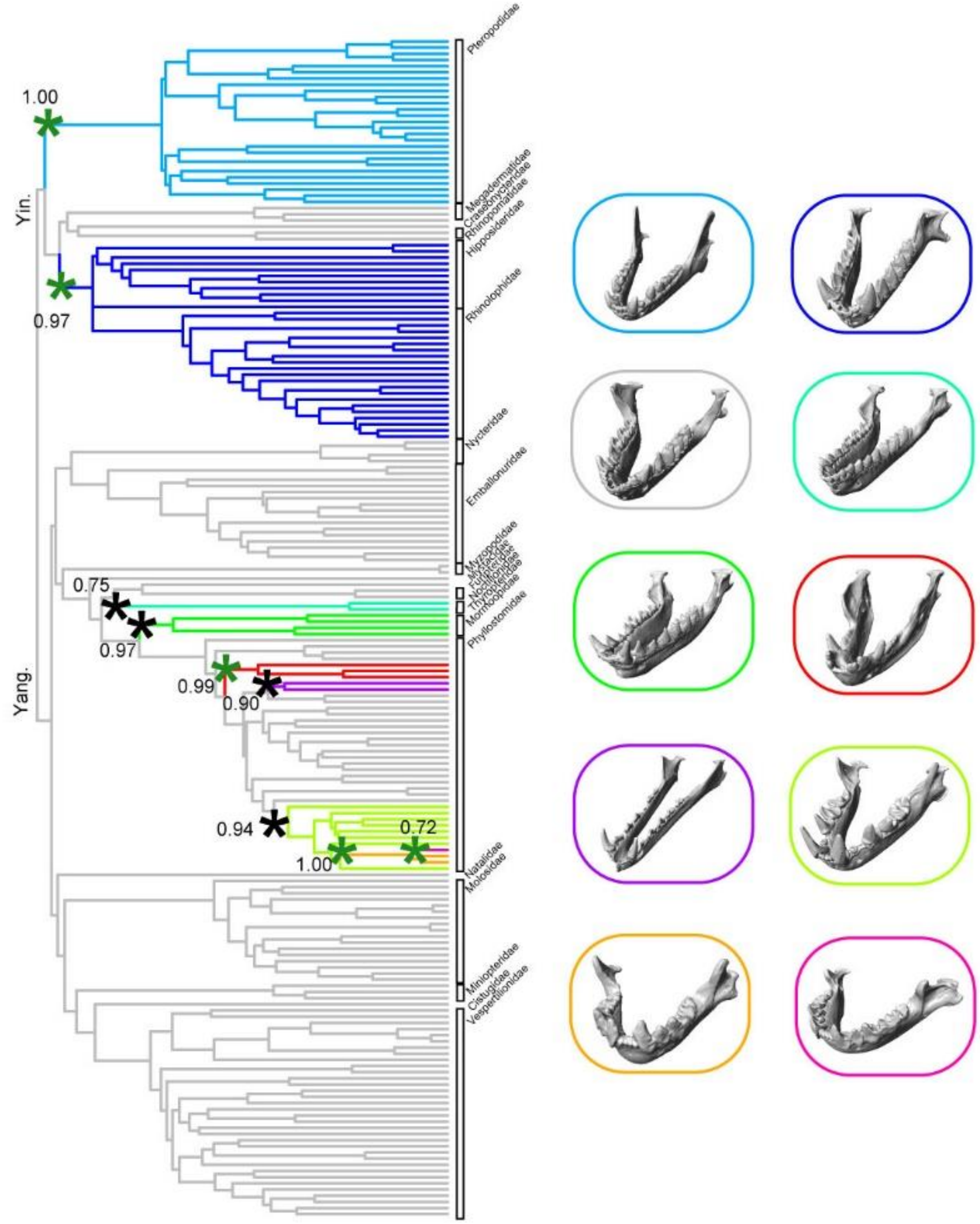

Supplementary Figure 14: Evolutionary shifts (*) in mandible shape across bats, as determined by "l1ou" adaptive landscape model fitting on PCA scores (PC 1-4; Fig. 1). Bootstrap support given for shift locations. Green shifts = also found in analyses of pPCA scores. Representative taxa from well supported shifts, from top to bottom; Left: *Rousettus aegyptiacus, Myotis lucifugus, Desmodus rotundus, Mormoops blainvillei, Choeronycteris mexicana, Sphaeronycteris toxophyllum*; Right: *Rhinolophus ferrumequinam, Thyroptera discolor, Chiroderma villosum, Centurio senex.* Yin. = Yinpterochiroptera. Yang. = Yangochiroptera.

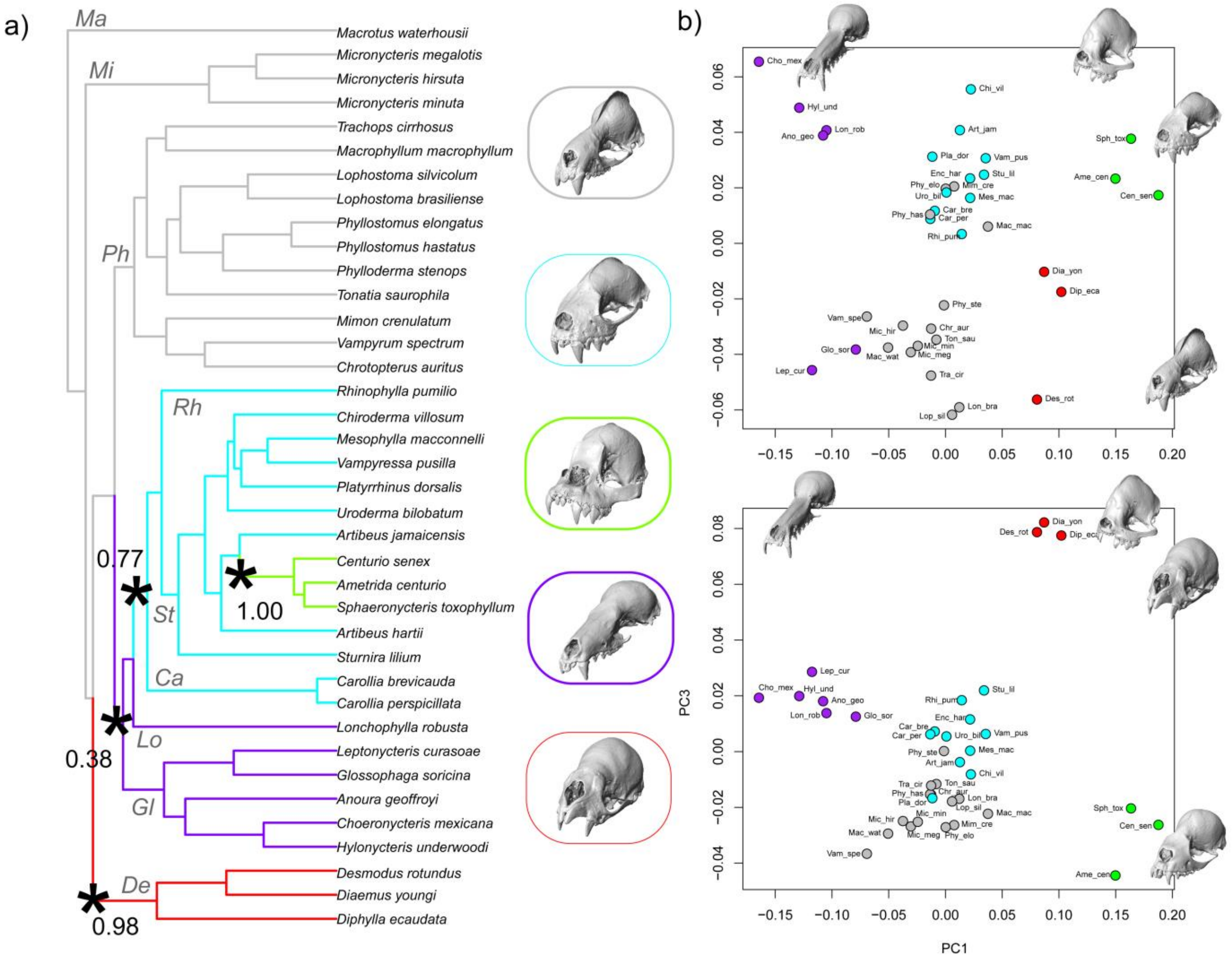

Supplementary Figure 15: Evolutionary shifts (*) in cranium shape in phyllostomids. A) Location of adaptive shifts, as determined by "l1ou" adaptive landscape model fitting on PCA scores. Bootstrap support given for shift locations. Representative taxa of well supported shifts, from top to bottom: *Lophostoma silvicolum, Artibeus jamaicensis, Ametrida centurio, Lonchophylla robusta, Desmodus rotundus.* Subfamilies as in Supplementary Figure 10. B) Cranial morphospace of phyllostomid bats based on PCA. Taxa illustrated: PC1 (+ *Centurio senex*, - *Choeronycteris mexicana*), PC2 (+ *Artibeus jamaicensis*, - *Lophostoma silvicolum*), PC3 (+ *Demodus rotundus*, - *Macrophyllum macrophyllum*). See source data file.

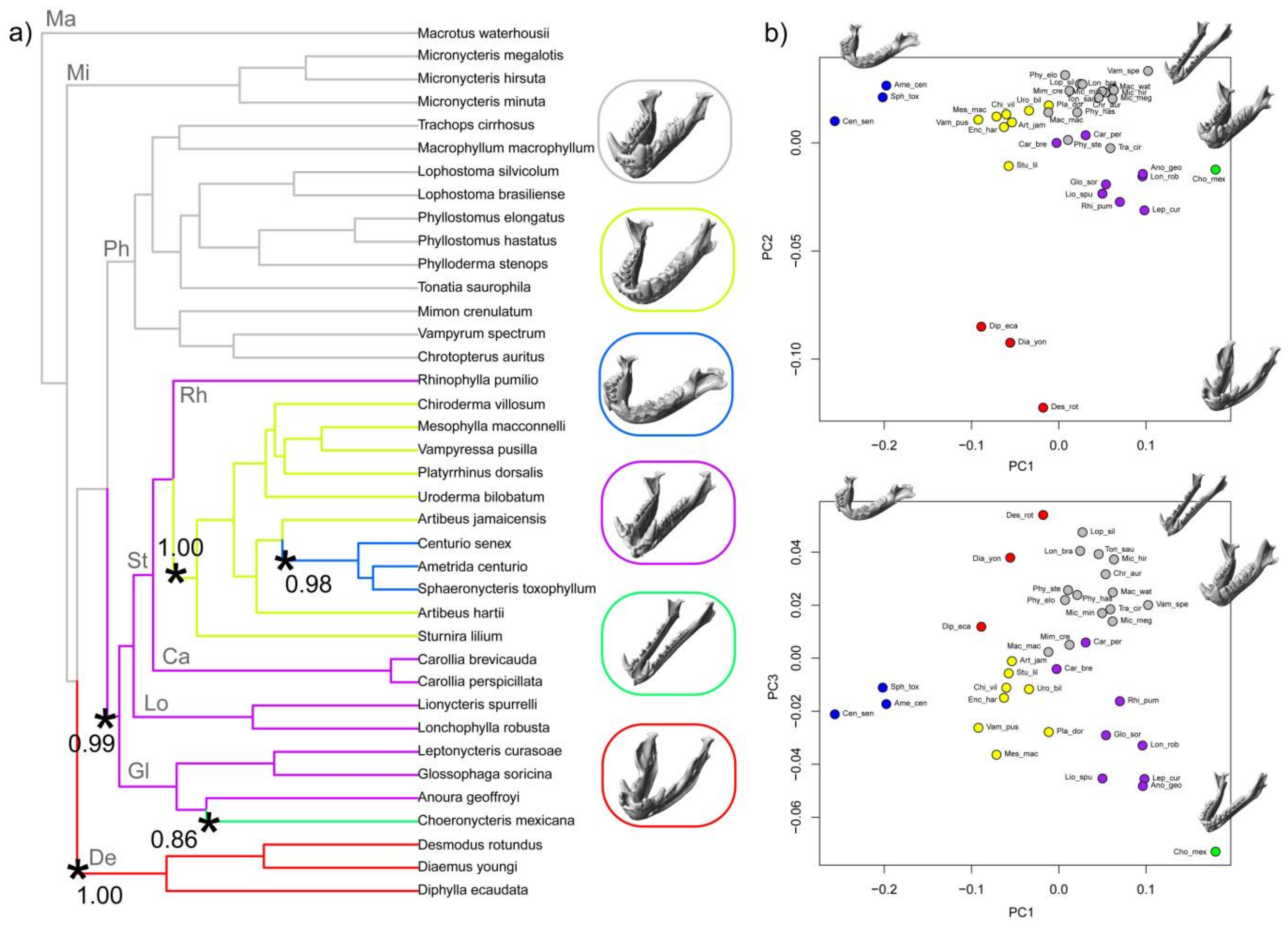

Supplementary Figure 16: Evolutionary shifts (*) in mandible shape in phyllostomids. A) Location of adaptive shifts, as determined by “l1ou” adaptive landscape model fitting on PCA scores. Bootstrap support given for shift locations. Representative taxa of well supported shifts, from top to bottom: *Trachops cirrhosus*, *Sturnira lilium* , *Centurio senex*, *Anoura geoffroyi*, *Choeronycteris mexicana*, *Desmodus rotundus*. Subfamilies as in Supplementary Figure 10. B) Mandibular morphospace of phyllostomid bats based on PCA. Taxa illustrated: PC1 (- *Centurio senex*, + *Choeronycteris mexicana*), PC2 (+ *Micronycteris hirsuta*, - *Desmodus rotundus*), PC3 (-*Lionycteris spurrelli*, + *Lophostoma silvicolum*). See source data file.

SUPPLEMENTARY TABLES

Supplementary Table 2: Parameters from l1ou model fitting from “l1ou” analysis for cranium and mandible shape evolution.

					PC1			PC2			PC3			PC4		
	regimes	pBIC	ΔpBIC		α	σ^2	llik	α	σ^2	llik	α	σ^2	llik	α	σ^2	llik
**cranium**																
**l1ou**	12	2854.96	148.96		0.431	16.4	-479.2	0.0736	5.36	-384.6	1.00E-07	8.87	-439.5	NA	NA	NA
**mandible**																
**l1ou**	16	3096.85	176.59		1.73	2.64E-03	4.34E+02	1.00E-07	6.37E-04	495	2.84E-01	2.75E-04	5.89E+02	1.04E-01	1.54E-04	6.35E+02

Supplementary Table 3: Results of mvMorph multivariate model fitting of cranium and mandible shape (from pPCA scores) evolution across phyllostomids. BM = brownian motion, OU = Ornstein-Uhlenbeck, EB = early burst. Multipeak OU models for dietary group (diet) were summarized over a distribution of 100 simmap character reconstructions, results are given as median (min, max). Bolded rows note the best supported model.

**cranium**	**regimes**	**k**	**loglik**	**AICc**	**ΔAIC**
BM	1	9	-297.23	614.2	96.58
EB	1	10	-297.23	616.61	98.99
OU1	1	15	-282.88	600.66	83.04
**l1ou**	**8**	**33**	**-211.78**	**517.62**	**0**
OU – Diet	7	30	-256.11 (-259.25, -252.83)	594.62 (588.07, 600.92)	77.00
**mandible**					
BM	1	5	-206.5	423.85	76.44
EB	1	6	-201.41	416.04	68.63
OU1	1	8	-206.5	431.14	83.73
**l1ou**	**5**	**16**	**-153.09**	**347.41**	**0**
OU-Diet	7	18	-170.00 (-171.63, -164.35)	388.01 (376.71, 391.26)	40.6

Supplementary Table 4: Results of mvMorph multivariate model fitting of cranium and mandible shape (from PCA scores) evolution across bats. BM = Brownian motion, OU = Ornstein-Uhlenbeck, EB = early burst. Multipeak OU models for dietary groups (diet) and echolocation emission type (EM) were summarized over a distribution of 100 simmap character reconstructions, results are given as median (min, max). Bolded rows note the best supported model. OU1 results are not given mvOU returned errors for this model for both the cranium and mandible.

**cranium**	**regimes**	**k**	**loglik**	**AICc**	**ΔAIC**
**l1ou**	**12**		**-1579.54**	**3346.83**	**0**
**OU-EM**	3	32	-1722.20 (-1730.68, -1717.51)	3511.12 (3501.75, 3528.08)	164.29 (154.92, 181.25)
**OU-DIET**	6	44	-1724.86 (-1780.42, -1718.10)	3542.90 (3529.40, 3654.03)	196.07 (182.57, 307.2)
**EB**	1	15	-1762.88	3556.36	209.53
**BM**	1	14	-1810.29	3649.11	302.28
**mandible**					
**l1ou**	**16**	**80**	**1445.99**	**3013.01**	**0**
**OU-DIET**	6	44	-1475.74 (-1496.50, -1467.11)	3044.99 (3027.73, 3086.51)	31.98 (14.72, 73.5)
**OU-EM**	3	32	-1510 (1527.72, 1508.61)	3086.89 (3084.11, 3122.34)	73.88 (71.1, 109.33)
**EB**	1	15	-1569.45	3169.54	156.53
**BM**	1	14	-1592.99	3214.54	201.53

Supplementary Table 5: Results of mvMorph multivariate model fitting of cranium and mandible shape (from PCA scores) evolution across phyllostomids. BM = Brownian motion, OU = Ornstein-Uhlenbeck, EB = early burst. Multipeak OU models for dietary group (diet) were summarized over a distribution of 100 simmap character reconstructions, results are given as median (min, max). Bolded rows note the best supported model.

**cranium**	**regimes**	**k**	**loglik**	**AICc**	**ΔAIC**
**BM**	1	9	-285.99	591.98	92.19
**EB**	1	10	-279.85	581.85	82.06
**OU1**	1	15	-285.99	606.87	107.08
**l1ou**	4	27	-214.106	499.79	0
**OU - diet**	6	30	-239.51 (249.46, -234.68)	560.43 (551.77, 581.33)	60.64 (51.98, 81.54)
**mandible**					
**BM**	1	9	-285.04	589.81	98.21
**EB**	1	10	-273.44	569.01	77.41
**OU1**	1	15	-284.71	604.33	112.73
**l1ou**	6	30	-204.59	491.6	0
**OU - diet**	6	30	-226.31 (-230.40, -216.25)	535.03 (514.92, 543.20)	43.43 (23.32, 51.6)

SUPPLEMENTARY REFERENCES

1. Arbour, J. H. & Brown, C. M. Incomplete specimens in geometric morphometric analyses. *Methods Ecol. Evol.* **5,** 16–26 (2014).

2. Gunz, P., Mitteroecker, P., Neubauer, S., Weber, G. W. & Bookstein, F. L. Principles for the virtual reconstruction of hominin crania. *J. Hum. Evol.* **57,** 48–62 (2009).

3. Arbour, J. H. & Brown, C. LOST: Missing morphometric data simulation and estimation. (2012). at <http://cran.r-project.org/web/packages/LOST/index.html>

4. Oba, S. *et al.* A Bayesian missing value estimation method for gene expression profile data. *Bioinformatics* **19,** 2088–2096 (2003).

5. Brown, C. M., Arbour, J. H. & Jackson, D. A. Testing of the Effect of Missing Data Estimation and Distribution in Morphometric Multivariate Data Analyses. *Syst. Biol.* **61,** 941–954 (2012).
