# Supplementary material for: Signatures of echolocation and dietary ecology in the adaptive evolution of skull shape in bats
Source: Nat Commun. 2019 May 2;10:2036. doi: 10.1038/s41467-019-09951-y (PMC6497661; doi:10.1038/s41467-019-09951-y)
Supplement: Supplementary file 2 — Reporting Summary [file 41467_2019_9951_MOESM2_ESM.pdf]

nature research

Corresponding author(s): Jessica Arbour

Last updated by author(s): 2019/3/25

Reporting Summary

Nature Research wishes to improve the reproducibility of the work that we publish. This form provides structure for consistency and transparency in reporting. For further information on Nature Research policies, see Authors & Referees and the Editorial Policy Checklist.

Statistics

For all statistical analyses, confirm that the following items are present in the figure legend, table legend, main text, or Methods section.

n/a	Confirmed	
☐	☒	The exact sample size (*n*) for each experimental group/condition, given as a discrete number and unit of measurement
☐	☒	A statement on whether measurements were taken from distinct samples or whether the same sample was measured repeatedly
☐	☒	The statistical test(s) used AND whether they are one- or two-sided<br>*Only common tests should be described solely by name; describe more complex techniques in the Methods section.*
☒	☐	A description of all covariates tested
☒	☐	A description of any assumptions or corrections, such as tests of normality and adjustment for multiple comparisons
☐	☒	A full description of the statistical parameters including central tendency (e.g. means) or other basic estimates (e.g. regression coefficient) AND variation (e.g. standard deviation) or associated estimates of uncertainty (e.g. confidence intervals)
☒	☐	For null hypothesis testing, the test statistic (e.g. *F*, *t*, *r*) with confidence intervals, effect sizes, degrees of freedom and *P* value noted<br>*Give P values as exact values whenever suitable.*
☒	☐	For Bayesian analysis, information on the choice of priors and Markov chain Monte Carlo settings
☒	☐	For hierarchical and complex designs, identification of the appropriate level for tests and full reporting of outcomes
☐	☒	Estimates of effect sizes (e.g. Cohen's *d*, Pearson's *r*), indicating how they were calculated

Our web collection on statistics for biologists contains articles on many of the points above.

Software and code

Policy information about availability of computer code

Data collection	Data was obtained from micro-CT scans of bat skulls, with landmark coordinates acquired using "Checkpoint" software. Phylogenies and ecological data were obtained from previous publications (citations provided).
Data analysis	Data was analyzed using published packages in R language, especially "phytools", "geiger", "mvOU", "l1ou", "geomorph" and "LOST". All necessary functions and packages are specified in the methods.

For manuscripts utilizing custom algorithms or software that are central to the research but not yet described in published literature, software must be made available to editors/reviewers. We strongly encourage code deposition in a community repository (e.g. GitHub). See the Nature Research guidelines for submitting code & software for further information.

Data

Policy information about availability of data

All manuscripts must include a data availability statement. This statement should provide the following information, where applicable:

- Accession codes, unique identifiers, or web links for publicly available datasets
- A list of figures that have associated raw data
- A description of any restrictions on data availability

Phylogenies are available through their respective publications (sources given). Source data files are available for all pPCA and PCA figures, DTT plots and missing data analyses (Fig. 1, 2, 6, S2-3, S6-12, S15-16) and for raw geometric morphometric data.

Field-specific reporting

Please select the one below that is the best fit for your research. If you are not sure, read the appropriate sections before making your selection.

☐ Life sciences ☐ Behavioural & social sciences ☒ Ecological, evolutionary & environmental sciences

For a reference copy of the document with all sections, see nature.com/documents/nr-reporting-summary-flat.pdf

Ecological, evolutionary & environmental sciences study design

All studies must disclose on these points even when the disclosure is negative.

Study description	Macroevolutionary analyses of bat skull shape, using model fitting and simulation approaches to study patterns of diversificiation from 3D shape data.
Research sample	Bat crania and mandibles from museum collections.
Sampling strategy	As a broad comparative study, our focus was on sampling widely across all bat families, from major ecological guilds within each family and including species describing the full morphological variation across each family where specimens were available.
Data collection	Bat skulls were micro-CT scanned, and 3D models were reconstructed using Mimics and Geomagic software. Landmarks (coordinates of key anatomical features) were collected using the program "Checkpoint".
Timing and spatial scale	No experimental sampling was conducted. Skulls were scanned from museum collections as available, and based on CT scanner access, from 2008 to 2017.
Data exclusions	No data were excluded.
Reproducibility	As no experimental procedures were used, we could not attempt to reproduce the results.
Randomization	Samples were not randomly allocated as they were assigned to their species in phylogenetic comparative analyses. Where feasible, simulated datasets were randomly generated to serve as a point of comparison for specific analyses.
Blinding	No blinding was used as specimens had to be assigned to specific species for phylogenetic comparative analysis.

Did the study involve field work? ☐ Yes ☒ No

Reporting for specific materials, systems and methods

We require information from authors about some types of materials, experimental systems and methods used in many studies. Here, indicate whether each material, system or method listed is relevant to your study. If you are not sure if a list item applies to your research, read the appropriate section before selecting a response.

Materials & experimental systems

n/a	Involved in the study	
☒	☐	Antibodies
☒	☐	Eukaryotic cell lines
☒	☐	Palaeontology
☐	☒	Animals and other organisms
☒	☐	Human research participants
☒	☐	Clinical data

Methods

n/a	Involved in the study	
☒	☐	ChIP-seq
☒	☐	Flow cytometry
☒	☐	MRI-based neuroimaging

Animals and other organisms

Policy information about studies involving animals; ARRIVE guidelines recommended for reporting animal research

Laboratory animals	No laboratory animals were used
Wild animals	All specimens were from previously accessioned collections. No wild animals were collected for this study.
Field-collected samples	The study did not involve field-collected samples.
Ethics oversight	The study did not involve any live animal sampling.

Note that full information on the approval of the study protocol must also be provided in the manuscript.
